# Seeing Bensheim’s refugee tent city: reflections on researcher- and respondent-generated photo-elicitation of the spatial dimensions of racial discrimination

**DOI:** 10.3389/fsoc.2025.1569958

**Published:** 2026-01-23

**Authors:** Claudia Ba

**Affiliations:** Department of Urban and Spatial Sociology, Technical University of Darmstadt, Darmstadt, Germany

**Keywords:** visual ethnography, photo-elicitation, racial discrimination, refugees, campization, space

## Abstract

This article explores the spatial dimensions of racial discrimination in a refugee camp in Bensheim, Hesse, Germany, using three photo-based methods. In response to the growing adoption of visual methodologies in refugee research, it critically examines the potential and limitations of each method, as well as contested academic assumptions that become visible through their combined application. I conceptualize photography as an embodied practice in the co-production of academic knowledge. Employing a layered framework, this article proceeds through three distinct methodological steps, examining in turn an aerial perspective using maps, researcher-generated photo-documentation during a “go-along” with a political representative (etic perspective), and auto-driven photo-elicitation with five refugees (emic perspective). The findings show how different perspectives on the tent city in Bensheim reveal the intersection of spatial dimensions of racial discrimination, including territorial stigmatization, peripheralization, and internal zoning. Photographs taken by female refugee respondents further emphasize embodied experiences of gendered and ethnicized discrimination within a space designed to contain and surveil “young Muslim men.” I emphasize the importance of researchers’ reflexivity regarding both epistemological frameworks and *locale* when employing photo-based methods.

## 1 Introduction

The visual appropriation of urban space is a long-standing—and contested—Eurocentric practice in the production of academic knowledge (Barwick-Gross et al., 2025). Visualizations of the built environment produce meanings that raise critical questions about who has the right to produce and show visual representations and who has the right to interpret them (Hall, 2013; Harper, 2012; Pink, 2001; Richard and Lahman, 2015). Since the reflexive turn, visual participatory methods have gained prominence across disciplines researching the nexus of migration, refuge, and spatial marginalization (Humpage et al., 2019; Moskal, 2019; Pauwels, 2015; Weber, 2019).^1^ This is especially evident in research involving female refugees, whose experiences are shaped by intersecting and multi-layered forms of disadvantage (Lenette and Boddy, 2013; Valiquette and Su, 2024). Visual ethnographers who employ joint photo-elicitation methods−meaning the inclusion of refugee or insider (emic) perspectives−tend to frame their methodological choice as inherently “participatory” and ethically superior to other photo-based approaches. This perception often rests on the assumption that these researchers give respondents “voice”—despite existing within unequal societal power structures (Pauwels, 2015). Certainly, these studies expose the disadvantages faced by refugees−highlighting the intersubjective accounts of sociopsychological distress caused by multi-layered vulnerabilities (Humpage et al., 2019; Weber, 2019).

However, there is still a significant lack of empirical research on racism in Germany (Wojnicka and Nowicka, 2024) and, in particular, of investigations using visual sociology approaches in migration, ethnic, and racial studies (Martiniello, 2017). To illustrate refugees’ appropriate accommodation, such as container-built Tempohomes (Misselwitz et al., 2022; Steigemann and Misselwitz, 2020), scholars in sociology, urban planning, and psychology have increasingly engaged with visual methods—including mental maps (Mehran et al., 2021; Moskal, 2019), architectural mapping, and researcher-conducted photo-documentation. In most of the latter cases, researchers use photographs of the built environment illustratively or only implicitly in the co-production of academic knowledge (cf. Misselwitz et al., 2022). Emic, or insider-informed, photographic research is virtually absent in academia, though the refugee activists of the Break Isolation Group do publish audio-visual *Lager Reports,* which include photographs (Barry, 2025; International Women Space, 2023). This article addresses the scholarly research gap by exploring visually refugees’ perceptions of spatial dimensions of racial discrimination: that is, the sociomaterial (re)production of exclusions through the interplay of the built environment and forms of human differentiation. Moreover, however extensive and moving existing studies may be, they do not address the interplay between conceptual and methodological assumptions, such as the academic intention and *locale*^2^ of photographing, the epistemology that frames photographic use, or the varying perceptions of the spatial dimensions of racial discrimination. This article seeks to contribute to both methodological and empirical debates by explicitly comparing different visual methods.

The article takes into account the *locale* and circumstances of refugees’ accommodation. Critical urban theorist Lipsitz (2007) emphasizes that dominant social and political actors use space to reproduce racialized power structures. Thus, tent-based refugee camps are a tool through which authorities control the mobility of refugees (Bulley, 2014). Historically, scholars studied refugee camps as a phenomenon occurring outside EU borders, yet the European Union’s increasingly restrictive asylum and accommodation policies (Kreichauf, 2018), as well as refugee camps’ “increasing urbanization” (Sanyal, 2012, p. 641), direct scholarly attention within EU borders.^3^ Existing studies on refugee housing in Germany provide valuable insights but predominantly focus on metropolitan areas such as Berlin (El-Kayed and Hamann, 2018; Grbac, 2013; Kreichauf, 2018; Mehran et al., 2021; Misselwitz et al., 2022; Schäfer, 2015; Steigemann and Misselwitz, 2020). Although these studies address the placement of encampments on peripheries and describe spatial exclusion and segregation from the inner city and institutions, there is little engagement with the refugees’ perception of the relationship of housing to spatial dimensions of racial discrimination. Further investigations into how refugee encampments function in medium-sized cities (Herslund and Paulgaard, 2021)—such as Bensheim—help to demonstrate the co-constitutive relationship between racial discrimination, accommodation, and spatial perceptions.

With this dual focus—on substantive and methodological issues—this article addresses the following research question: “What are the potentials and limitations of various photo-based methods in the co-production of academic knowledge about the spatial dimensions of racial discrimination in tent-based refugee accommodation?” In the remainder of the introduction, I provide contextual background on the tent city used as a case study (1.1), outline my conceptual framework of spatial dimensions of racial discrimination (1.2), and discuss my methodological assumptions, particularly concerning the use of photography (1.3). In section 2, I introduce and critically review three photo-based methods, addressing their capacity to capture and represent experiences of discrimination and their validity in sociological research. They are aerial imagery, researcher-conducted photographs (representing an outsider, etic, perspective), and participant-generated photographs presenting the refugees’ insider (emic) perspective. In Section 3, I present the analytical outcomes from applying these three methods in Bensheim, Germany, during field research conducted along with the master’s student Yasmin Eismayr, who established initial contact with political representatives and social workers from the German Red Cross and assisted in data collection and transcription. While we conducted the data collection jointly and the analysis was partially collaborative, it ultimately constitutes a personal contribution for the purpose of this article. When referring to joint fieldwork and assumptions at various stages of research, I make use of “we” or “us.” In instances where conclusions are my own, I explicitly indicate it, using “I” and “me.” This section presents a chronological, step-by-step account, highlighting the data collection and interpretive process at each stage of the visual ethnography, weighing their potentials and limitations.

### 1.1 Context: the emergence of the tent city

Kreichauf (2018) shows that Europe’s asylum policies became more restrictive after 2015, making mass accommodation in camps, using rapid building techniques, a widespread practice. German politicians defended lightweight accommodation due to their financial feasibility and “temporary” nature—echoing the rationales of discount retail architecture (Spiegel, 2015). Tent cities, Tempohomes, and container villages emerged as political responses, prompting adjustments to the German Federal Building Code. As Kreichauf (2018, p. 11) notes, “Section §246, Articles 8–13 [enabled] the location of accommodation in industrial and commercial areas, the exemption from building and use requirements, the conversion of office buildings and warehouses to refugee shelters, and the installation of mobile structures such as tents and containers.”

**Figure 1:**
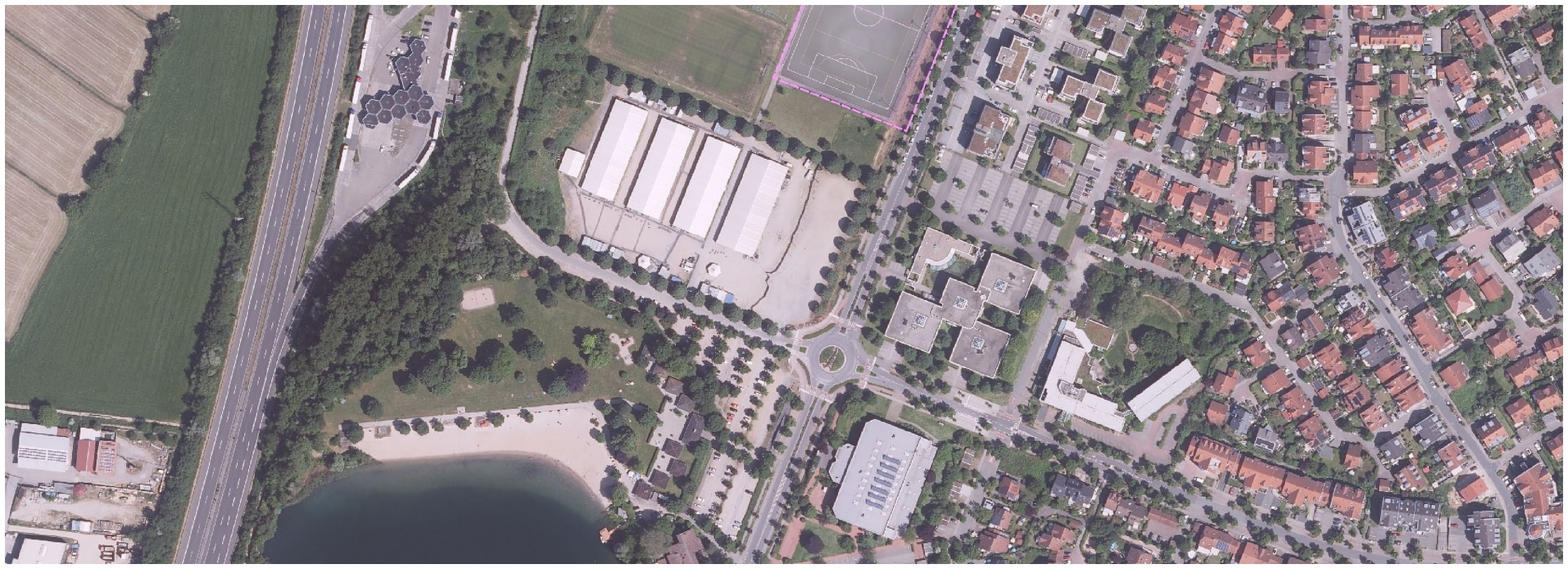
Aerial perspective of the tent city at the periphery of Bensheim. Map by Zentrale Kompetenzstelle für Geoinformation beim Hessischen Landesamt für Bodenmanagement und Geoinformation. Geoportal Hessen.de. Der Kreisausschuss des Kreises Bergstraße, Organization GDI-Südhessen (2024-07-08) Kommunale Boden- und Raumplanung Kreis Bergstraße (56856) “Data license Germany—attribution—Version 2.0” at www.govdata.de/dl-de/by-2-0.

**Figure 2:**
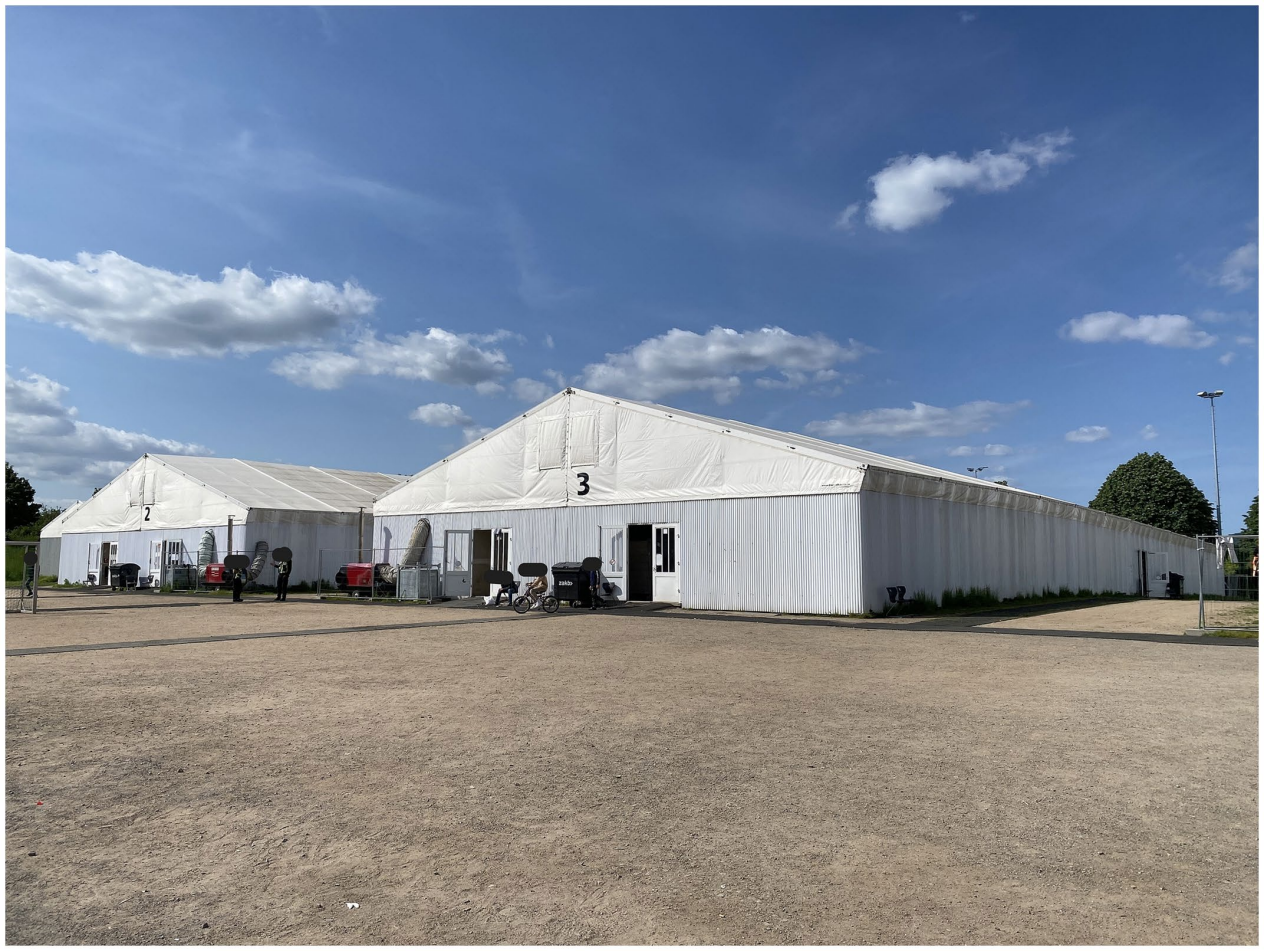
View on the inhabited tents and the vast sand “piste,” photo-documented by Eismayr (2023).

**Figure 3:**
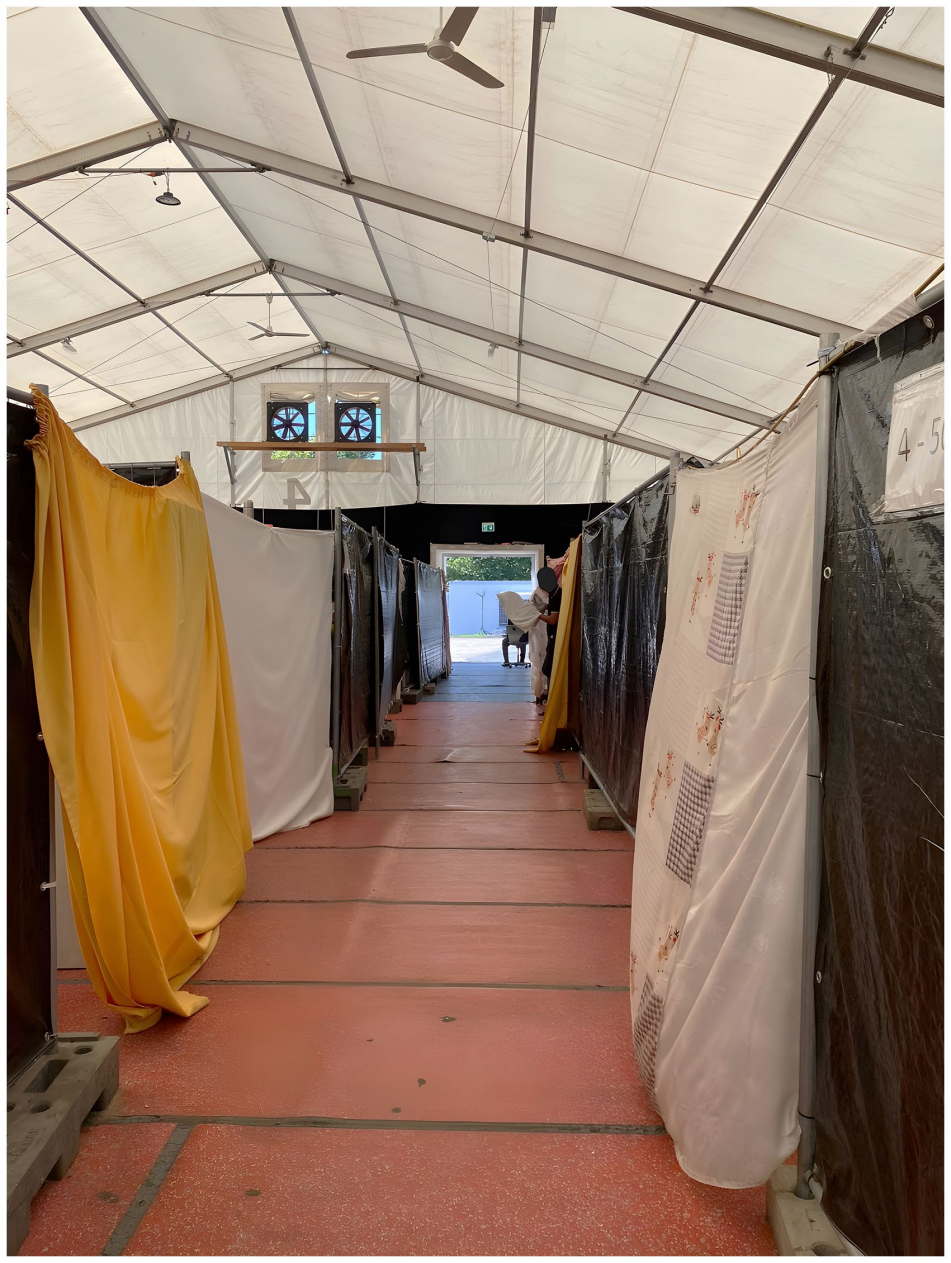
Habitation units separated by construction fences, tarpaulins, and bed sheets, photo-documented by Ba (2023).

The federal allocation “key” (the *Königsteiner Schlüssel*) proportionally assigns individual asylum seekers across the German federal states (Bormann and Werner, 2024). States apply additional constraints on duration and place of residence through “abode constraints” (*Wohnsitzauflagen*) (El-Kayed and Hamann, 2018). This decentralized system places the responsibility for refugee housing on local politicians rather than the federal government (El-Kayed and Hamann, 2018). Thus, at the state level, the Hessian Ministry for Economic Affairs, Energy, Transport, and Housing followed the federal argument and ruled that the building regulations for collective accommodation housing more than 30 asylum seekers operate differently from buildings with residential use, because “residential use is characterized by a permanent, self-directed household management and the voluntary nature of residence (trans. by CB) (HMWEVW, 2022).” In contrast, these special-purpose buildings (*Sonderbau*) for mass accommodation only meet a “temporary accommodation need (trans. by CB).” Hesse’s practical response was the establishment of tent-based accommodation, for which the national newspaper FAZ (2015) labeled the federal state the “land of tents.”^4^ The municipal administration of Bensheim-Auerbach established a so-called “tent city”^5^ on a fairground because the site met the requirements for infrastructural provision, such as wastewater and the supply of fresh water and electricity. In Bensheim, two large festival tents were rented, which were subdivided into different compartments using construction fences and were designed to accommodate 300 people each (Figures 1–3).

Established in response to rising numbers of refugees and asylum seekers after 2015, the Bensheim tent city operated in two main phases, from 2015 to 2019 and from 2022 to 2024. The multi-ethnic camp reached its maximum capacity of 1,000 individuals in 2022, following the Russian invasion of Ukraine, while refugees from “third” countries such as Afghanistan, Syria, Türkiye, and Iran continued to arrive in high numbers (Alfter, 2023; Ganster and Kaufhold, 2023). Two years later, in September 2024, the municipal administration closed the tent city due to declining asylum applications—in part because the German government designated safe countries of origin and the subsequent decrease in arrivals (Gerritz, 2024). This article presents empirical material based on fieldwork conducted during the summer of 2023, when refugee numbers had stabilized but remained high—approximately 500 people lived in the camp at that time.

### 1.2 Conceptual framework of spatial dimensions of racial discrimination

In this section, I outline the conceptual framework, showing how scholarly perspectives on the spatial dimensions of racial discrimination informed my assumptions and choice of methods. My symbolic interactionist perspective lays the basis for conceptualizing racial discrimination as a perceived and performed interaction with people, objects, and architectures. This approach combines Hirschauer (2021, 2023) concept of human differentiation and Steets (2015) work on the meaningful construction of the built environment.

I frame my analysis around the spatial dimensions of racial discrimination. Race as a social construct represents an abstraction from broader social realities, and its conceptualization varies significantly across historical and cultural contexts (Clair and Denis, 2015; Delaney, 2002). In particular, the German term *Rasse* differs significantly from US-American discourses on “race.” Alexopoulou (2019) and Löw (2023) further elaborate on divergent usages of race in the German language. German-speaking academic debate was previously largely devoid of “race” (i.e., *Rasse*) after the 2nd World War, whereas English academic engagement advanced with the term from “[its first usage] to describe peoples and societies in the way we now understand ethnicity or national identity” (Clair and Denis, 2015, p. 857). Balibar and Wallerstein’s (2019) argument about neo-racism in Europe is central to current German academic discussions: they claim that racism now operates “without race.” By this, they mean that “racial” categorizations instead work along cultural or religious lines and are no longer solely based on essentialized biological understandings of race (Bojadzijev, 2015). In Germany, the failure to address its racial and colonial past persists until today, leading to the inability to frame Germany as a “post-migrant society” and to recognize the present-day reality of a diversified society (El-Tayeb, 2016).

Wider sociological debates have evolved around the issue of the extent to which race can be analytically subsumed under the concept of ethnicity (Löw, 2023; Wacquant, 2023). Wacquant (2023) argues that it could be subsumed, rendering the concept of race epistemologically redundant. In contrast, Löw (2023) contends that race remains an empirically relevant and lived social reality, as individuals continue to categorize themselves and others along racial lines. According to her, race constitutes an irreducible empirical concept that sociologists must address in social research. However, in refugee studies in Europe, some scholars criticize the predominant focus on *ethnic* minorities rather than on race and racism (Kappelhof and De Leeuw, 2019). They argue that the emphasis on ethnicity arises from the perception that refugees in Europe exhibit diverse “cultural differences, language barriers, [and] sociodemographic characteristics” (Kappelhof and De Leeuw, 2019, p. 117), which scholars cannot easily attribute to “race.” Similarly, Wacquant (2007, p. 68) argues that stigma in Europe is less frequently attributed to phenotypical characteristics (such as skin color)—as is often the case for African Americans in the United States—and more commonly linked to perceived markers of ethnicity, class, habitus, and religious identity. The situation is clearly complex: Castro Varela and Mecheril (2016) and El-Tayeb (2011) have demonstrated that in Europe, intersecting social categories—such as “Muslim,” “young,” “men”—are frequently mobilized to legitimize collective fears and even violence directed at these groups, showing how differentiations along racial and ethnic lines are blurred.

Thus, there is both empirical evidence for the social constitution of race in Europe and considerable conceptual messiness over “race” and “ethnicity,” with consequent methodological problems in operationalizing these terms. For clarity, in this article, I follow Clair and Denis (2015), who conceptualize race both as socially (re)produced and as an experienced and lived reality, and racial discrimination as the unequal treatment of these (constructed) races. I argue that racial discrimination is spatially and materially enacted. Racial discrimination within a specific context points toward social processes of unequal treatment in its making. Thus, I am not looking at the segregation of refugees as a societal fact but at how their segregation is (re)produced.

How can I conceptualize spatial dimensions of human differentiation and thus of racism? Architecture not only serves as a means of physical construction but also as a tool of differentiation, which stratifies hierarchies and inequalities. In what follows, I briefly set out Hirschauer’s (2021, 2023) heuristic of human differentiation and provide examples of spatial manifestations of human differentiation.

The heuristic outlines a sequence of four levels: (1) differentiation, (2) categorization, (3) dissimilation, and (4) classification (Hirschauer, 2023, pp. 75–80).

1. *Differentiation* emerges from everyday observational practices. It helps to explain what Bourdieu referred to as “taste,” or a form of attunement or aversion toward particular people and objects. However, differentiation remains ambiguous and often results in “leftover categories (trans. by CB),” which may reinforce rather than deconstruct social boundaries (Hirschauer, 2023, p. 76).
2. *Categorization* is the next level, where internalized differentiations are treated as societal facts—such as the dichotomy between Deutsch and Undeutsch (i.e., “German” and “un-German”). At this stage, as Hirschauer (2023, p. 77) notes, “the unequal positioning of *the* Other renders the formation of a shared category meaningless (trans. by CB).” Collective identities, stereotypes, and prejudices emerge at that stage.
3. At the level of *dissimilation*, categorizations shape concrete interactions. People translate social categories into differentiated practices—for example, women are employed, trained, or addressed differently from men, thereby becoming physically and socially distinct. Thus, humans enact a “material widening of distance” through practices (Hirschauer, 2023, p. 78).
4. Finally, *classification* formalizes dissimilation. It involves systems such as the nation-state, with standardized language policies, or the delineation of territorial boundaries (Hirschauer, 2023, p. 79). Through these mechanisms, the ambiguity of individuals and groups is reduced, and they are assigned stabilized identities based on formalized criteria.

Hirschauer’s model emphasizes three key principles that underlie these processes: the agential role of the “objects” of differentiation (in the sense that people always co-construct their own identities); their relational nature (each act of categorization implies a counterpart); and their perspectivity (attributing always involves questions of authorship). Who assigns differences? Asymmetries become solidified when such distinctions are not merely descriptive but evaluative—when they imply who is “better/worse, superior/inferior” (trans. by CB) (Hirschauer, 2023, p. 87).

Let me now bring Hirschauer’s heuristic into dialogue with the sociological framework of the built environment developed by sociologist Steets (2015). Steets’ model offers a valuable lens to understand the built environment as a product of social processes. She builds on the work of Thomas Luckmann and Alfred Schütz, arguing that objectifications—such as tent-based settlements—represent an externalization of societal structures, for example, federal and EU regulations. In turn, society internalizes this material order, which then becomes co-constitutive of the social order, such as the multiple exclusions of refugees through accommodation practices. Once constructed, society perceives the built environment as a fact, and it thus reinforces the very ideas that shaped it. While Steets (2015) approach is productive for interpreting the symbolic significance of architecture, it does not explicitly engage with how the built environment architecturally and spatially enacts racial exclusion and other social inequalities. To take this further conceptual step, I adopt a relational understanding of space: humans produce spaces, including their material and concrete forms, through discursive representations and societal processes, which are therefore co-constitutive in shaping societal conditions (Belina and Michel, 2019; Löw, 2008). By combining relational space with Steets’ framework, I conceive the built environment as neither ahistorical nor devoid of signifiers, but rather as “an embodiment of power, class, gender, and race relations” (Brown, 2019, p. 1). By integrating Steets (2015) approach with Hirschauer (2023), I offer a more nuanced understanding of how human differentiations are sociomaterially reproduced. Consequently, my focus lies on the interactions and situatedness of racialized perceptions and practices with and through the built environment (De Certeau, 1985; Löw, 2008). This synthesis holds analytical value for ethnographic research, as it allows us to trace how symbolic and spatial orders interact in the lived experiences of racialized subjects.

The following fourfold model is my contribution to a sociomaterial approach to understanding human differentiation:

1. *Spatial differentiation.* Architectural styles evoke associations—such as taste, aversion, or esthetic attunement. These affective responses reflect everyday cultural practices but still leave room for multiple spatial interpretations and usages, so at this level, materials and architectural elements do not possess fixed and predetermined meanings. Rather, architectural objects can be deployed flexibly and acquire new significance depending on their context. *Spatial differentiations* are best illustrated by the perceived atmospheres of spaces, e.g., poorly lit underpasses fail to consider gendered safety needs, rendering them particularly exclusionary for women, leading to strategies of avoidance. Or, taking the example of wire mesh fencing in the tent city, this was originally intended for industrial or construction site enclosures. Praised for its lightweight structure and low cost, this material gains new meaning in the context of refugee encampment. In this setting, wire mesh fencing signals the provisional and makeshift nature of human containment and serves as a tool for controlling mobility and organizing social life. It demarcates boundaries between private, semi-public, and public zones and enforces waiting, such as by closing off kitchen areas outside of mealtimes. In this case, a single material can exceed perceptions of atmosphere and cross all four levels of differentiation, manifesting and negotiating social order through its flexible, yet symbolically and politically charged, deployment in space. Yet, the fencing is reusable on construction sites.
2. *Spatial categorization* attributes fixed functions to architectural forms—for example, banlieues or forms of zoning. In such spaces, humans inscribe and reify attributions or stereotypes toward the inhabitants such that, for example, banlieues suggest delinquent behavior. Spatial categorization forecloses alternative meanings or potential redefinitions of space and seeks to abolish ambiguity. Empirical studies of reception centers and refugee camps state that internal zoning and designed lack of privacy significantly contribute to experiences of psychological stress (Kreichauf, 2018; Mehran et al., 2021; Misselwitz et al., 2022). Humans inscribe categorizations in refugee encampments spatially, such as hierarchies within the refugee community that result from “zoning” (Mehran et al., 2021). Zoning is extremely complex and touches multiple stereotypes and prejudices of human differentiation, which work along lines of age, gender (e.g., male/female toilets), and/or ethnicity (e.g., the same ingroup shares compartments within the larger tents).
3. *Spatial dissimilation* extends beyond external ascriptions by shaping interactions. At this level, it forecloses action so that humans not only categorize people from an outsider perspective (e.g., as refugees) but also treat them accordingly, e.g., the police will more likely racially profile refugees. Here, Wacquant’s concept of “territorial stigmatization” (2007) is applicable, as it regulates not only performances within spaces such as a camp but also persists outside them. He underlines that a specific *locale* can temporarily fix a “social purgatory,” stigmatizing body, character, nationhood, or the religion of its inhabitants (Wacquant, 2007, p. 67). This stigma can also exceed the spatial confines of refugee encampments and influence other urban contexts, including the housing market (El-Kayed and Hamann, 2018). Thus, *spatial dissimilation* prescribes social realities in and outside the built environment of the camp.
4. *Spatial classification* denotes architectural forms that explicitly encode and stabilize the social stratification inherent in *spatial dissimilation*. These environments, and the human assignments they dictate, work to eliminate ambiguity and reinterpretation. Societies and institutions spatially allocate refugees to temporary, lightweight camps in designated territories. Such mechanisms institutionalize dissimilation, embedding it within a repetitive and standardized built environment that extends across Europe and beyond its borders.

Building on this conceptualization of racial discrimination and its spatial manifestations, I formulated three distinct assumptions that informed my decisions concerning field access, methodological approach, and the scope of my empirical engagement:

- Delaney (2002) and Kreichauf (2018) emphasize the dichotomous construction of refugee encampment in opposition to the host society. Both physical barriers—such as fences—and the placement of refugee camps on urban peripheries and under surveillance, along with restricted access to resources and social contacts, serve to reinforce this division (Grbac, 2013). I assumed that I could observe a stratified process of racialization manifesting through the materials and architectural elements used. Based on this initial assumption, I concluded that it was necessary to first describe the encampment before making contact with the refugees within it.
- To find empirical proof of racial discrimination, I further assumed that it is less grounded in explicit references to race and more in markers of ethnicity, religion, or gender. The spatial enactment of multiple and intersecting forms of human differentiation thus required an open and attentive approach. I chose not to specifically ask refugees about race and racial discrimination to avoid introducing these preconceived ideas. However, I assumed that everyday spatial routines within the camp were shaped by spatial control and restriction, that spaces were subdivided functionally (e.g., areas designated for eating or sleeping) and through categorization of specific groups—such as refugees and staff, women and men, or individuals differentiated along ethnic or religious lines. I also assumed that while some camp residents perceive spatial arrangements as potentially conflictual along racial lines, they do not attribute xenophobic meaning to all of them.
- I assumed that public spaces take on the function of alternative spaces of privacy for refugees: that leaving the camp premises was a coping strategy, a way of seeking relief from the cramped living conditions and lack of personal space. I asked myself about the role ascribed to public environments—such as hiking trails, markets, tourist sites, and streets—which potentially emerge as alternative places of rest, reflection, and solitude. The literature suggests that mental or cognitive mapping exercises could pose challenges for participants, both in terms of navigation and the abstraction involved in visually representing spatial relationships (see Buhr, 2021). For this reason, I opted for a semi-participatory method involving self-produced photo-documentation, in which refugee respondents^6^ themselves would produce photographs. Although I specifically invited them to document their living conditions within the tent city and everyday life, I was surprised to find that they took most of the photos outside the camp premises.

In the following section, I aim to elucidate the rationale behind the choice of photographic methods and their situatedness within refugee research.

### 1.3 Epistemic situatedness of photography

**Figure 4:**
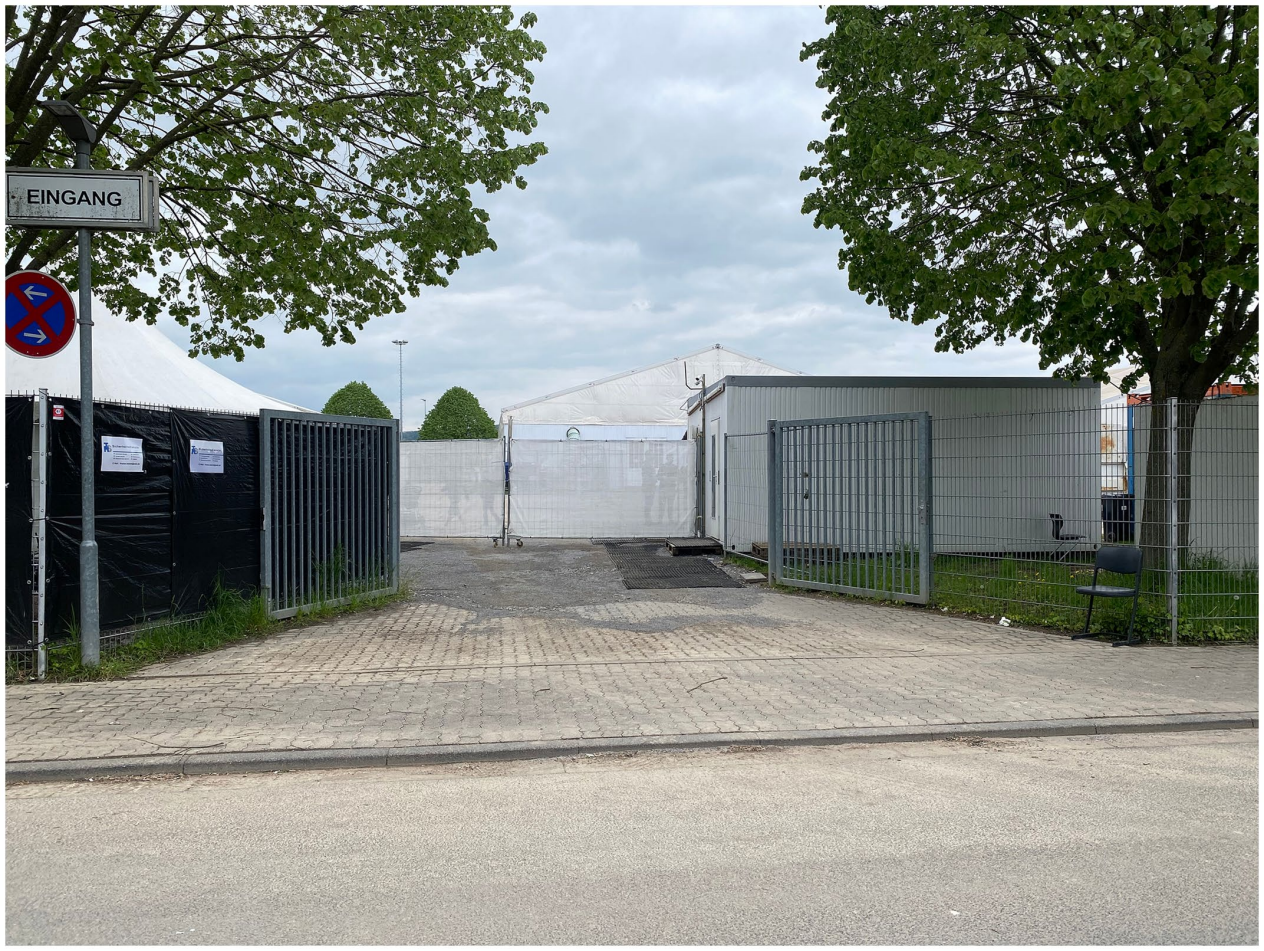
The entrance of the refugee “tent city” of Bensheim, photo-documented by Eismayr (2023).

**Figure 5:**
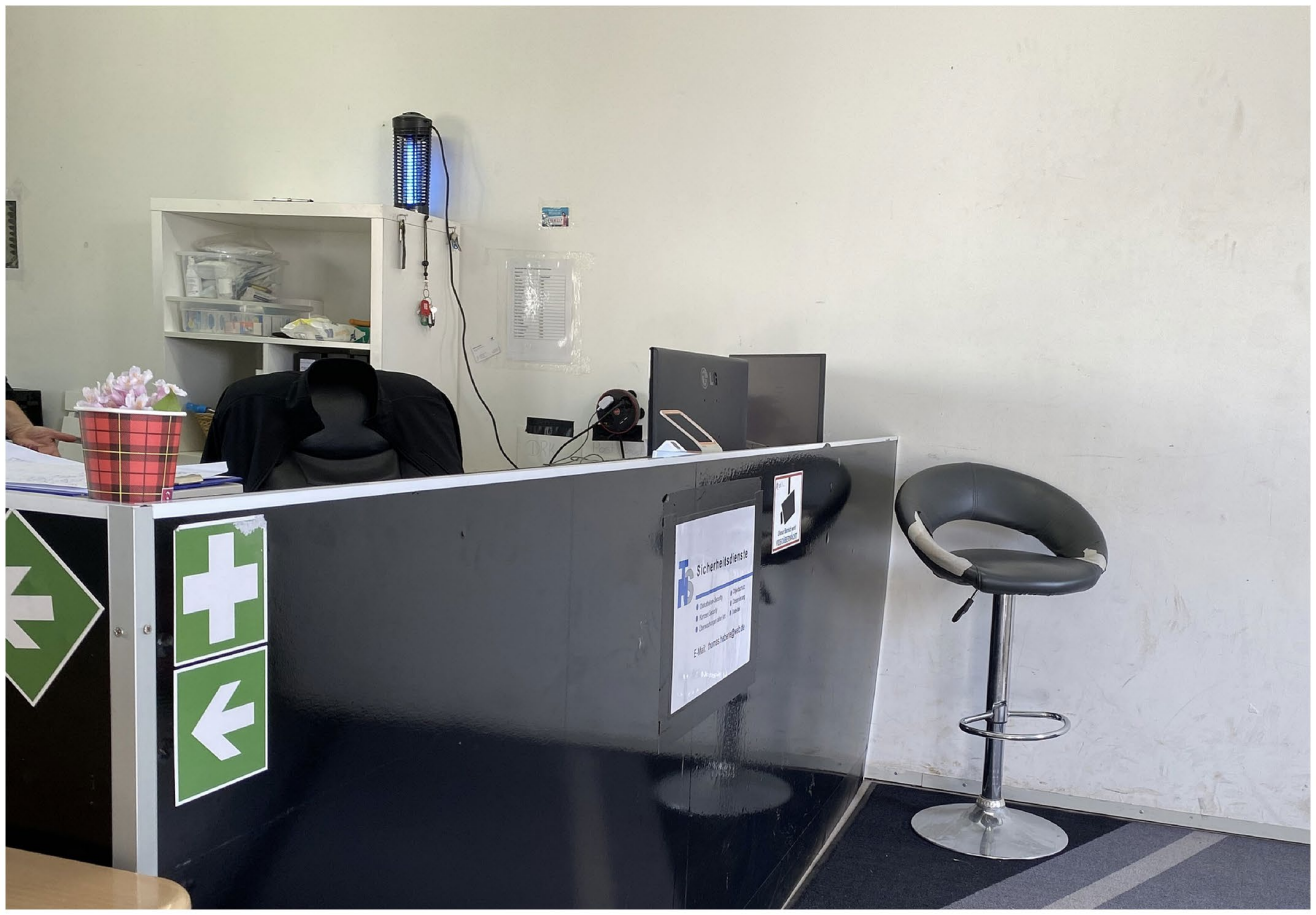
The entrance area inside the container, equipped with fingerprint scanners, photo-documented by Eismayr (2023).

**Figure 6:**
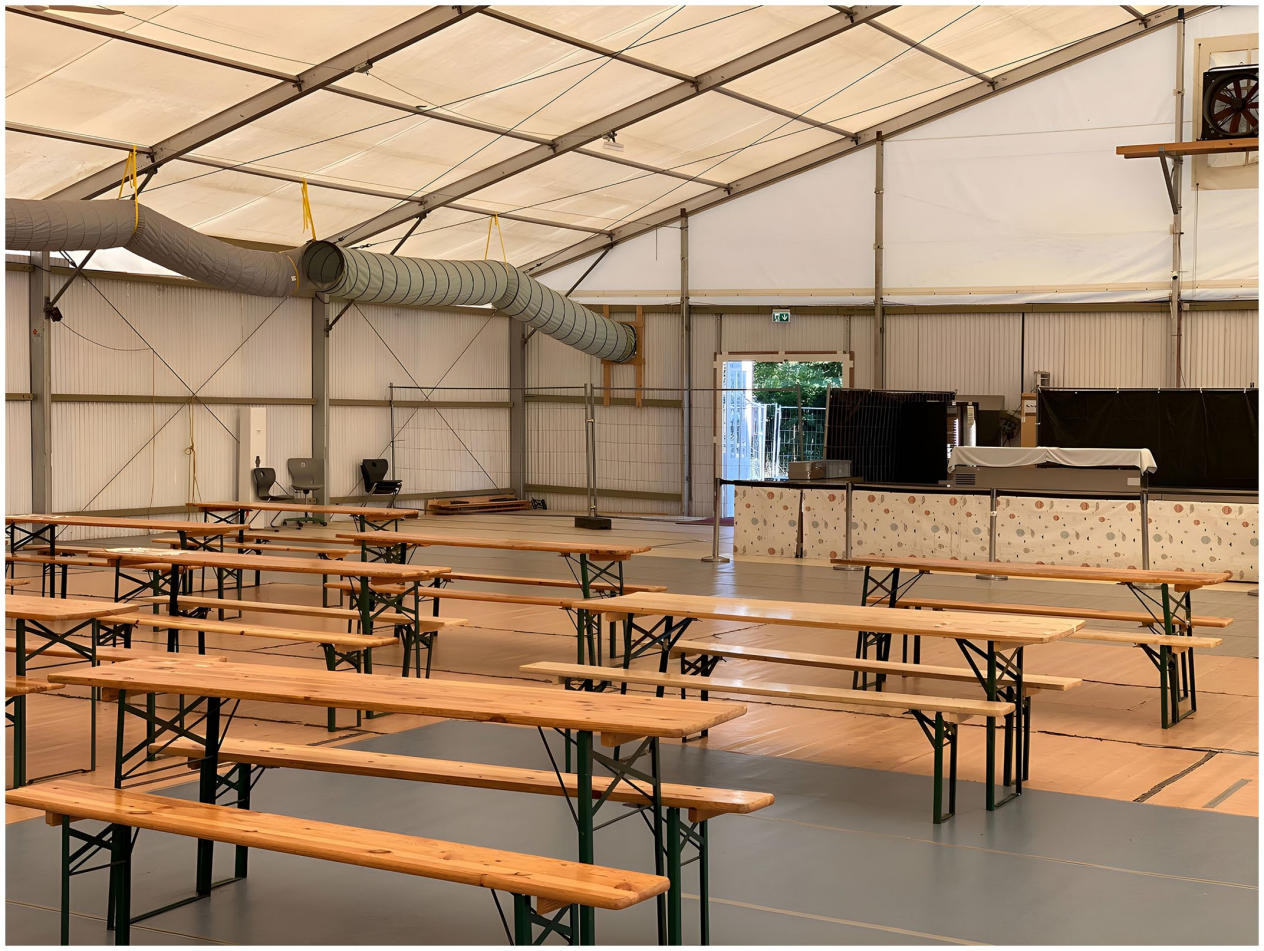
Food distribution area inside the social tent, photo-documented by Eismayr (2023).

**Figure 7:**
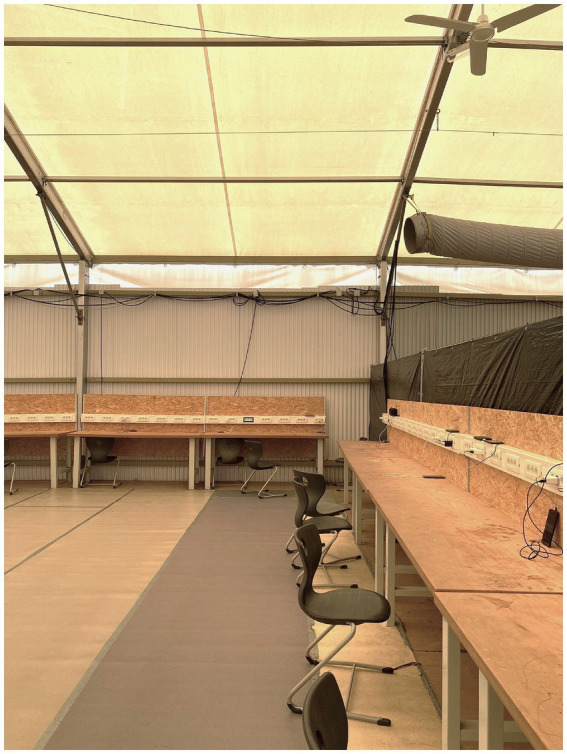
The social tent and power strips to charge mobile devices centrally, photo-documented by Eismayr (2023).

**Figure 8:**
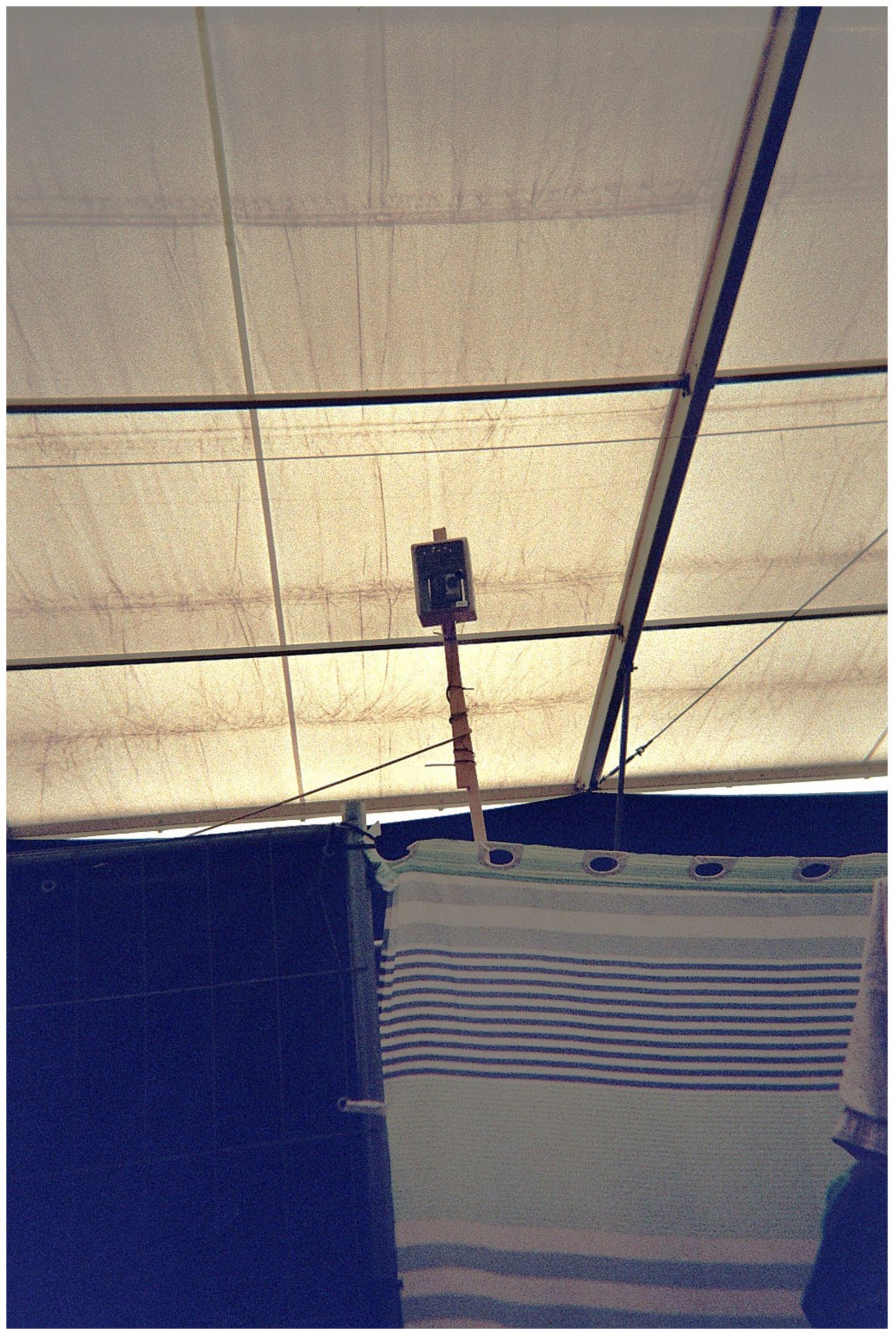
A device for measuring temperatures that Amina mistook for a camera, photographed by Amina (2023).

**Figure 9:**
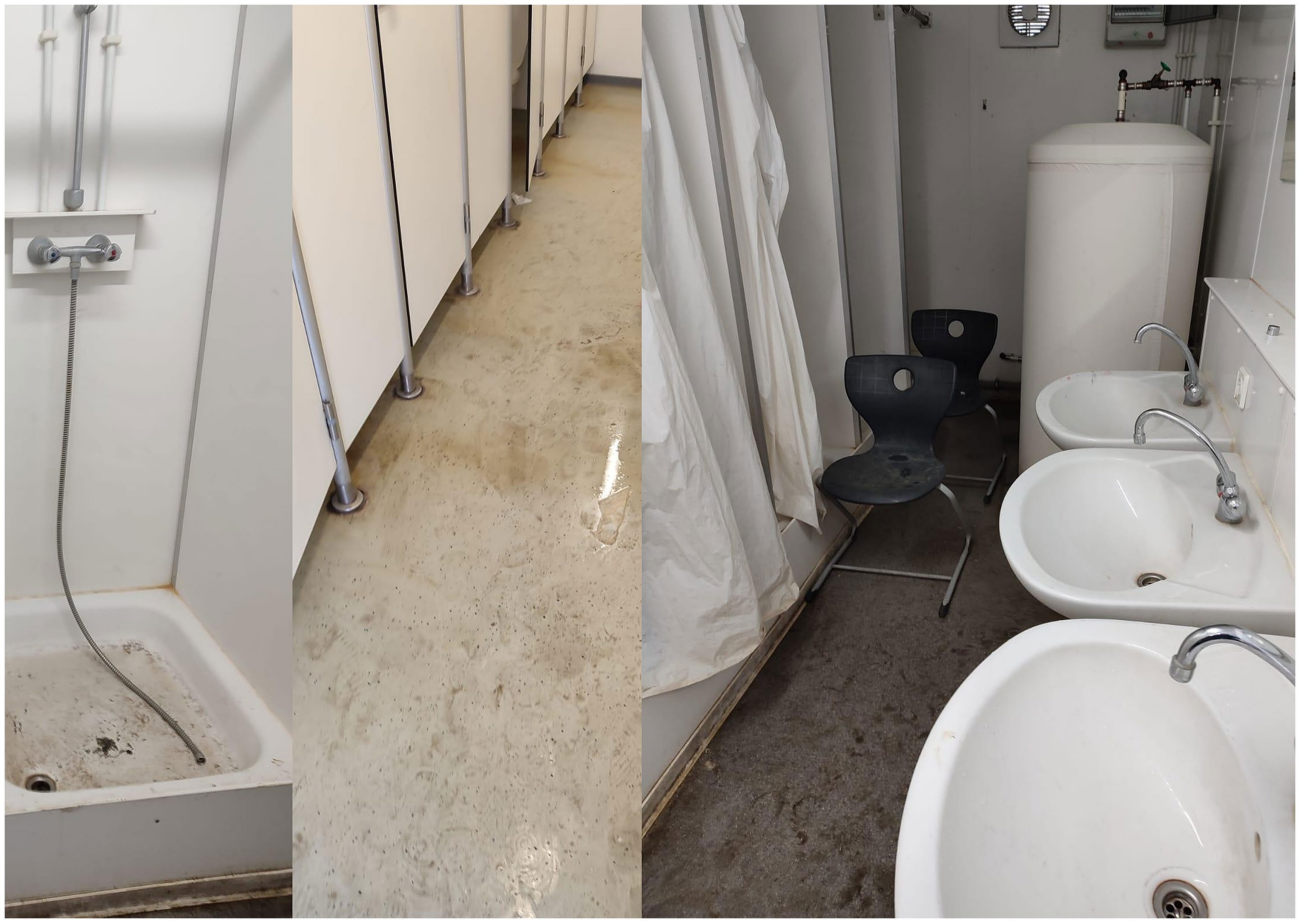
Shared sanitary facilities in a container, photographed by Maria (2023).

**Figure 10:**
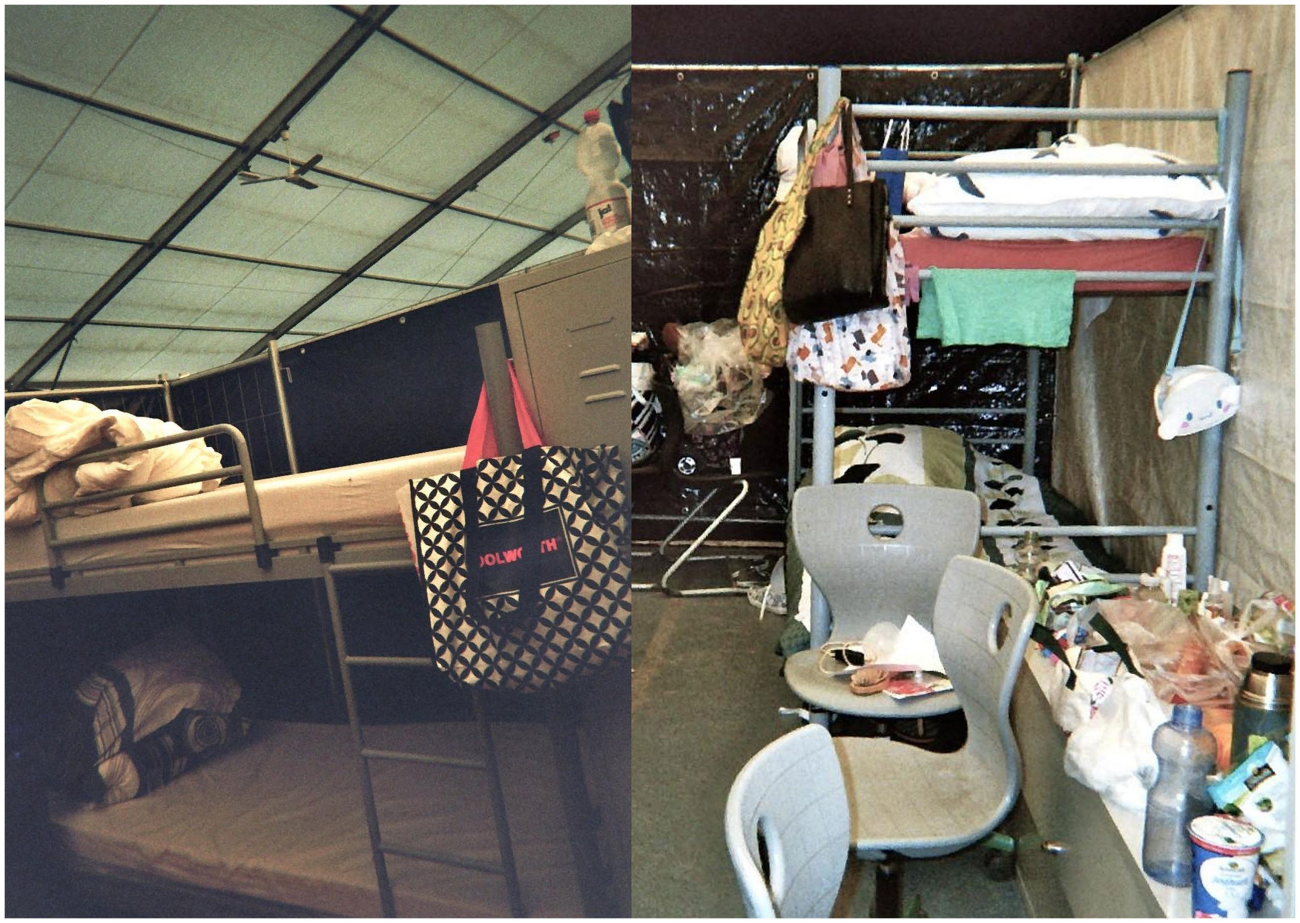
Bunk beds inside the divided sleeping units. Left: Amina’s upper bunk bed with personal belongings stored in her bag, photographed by Amina (2023). Right: Maria sharing two bunk beds with her two children, photographed by Maria (2023).

**Figure 11:**
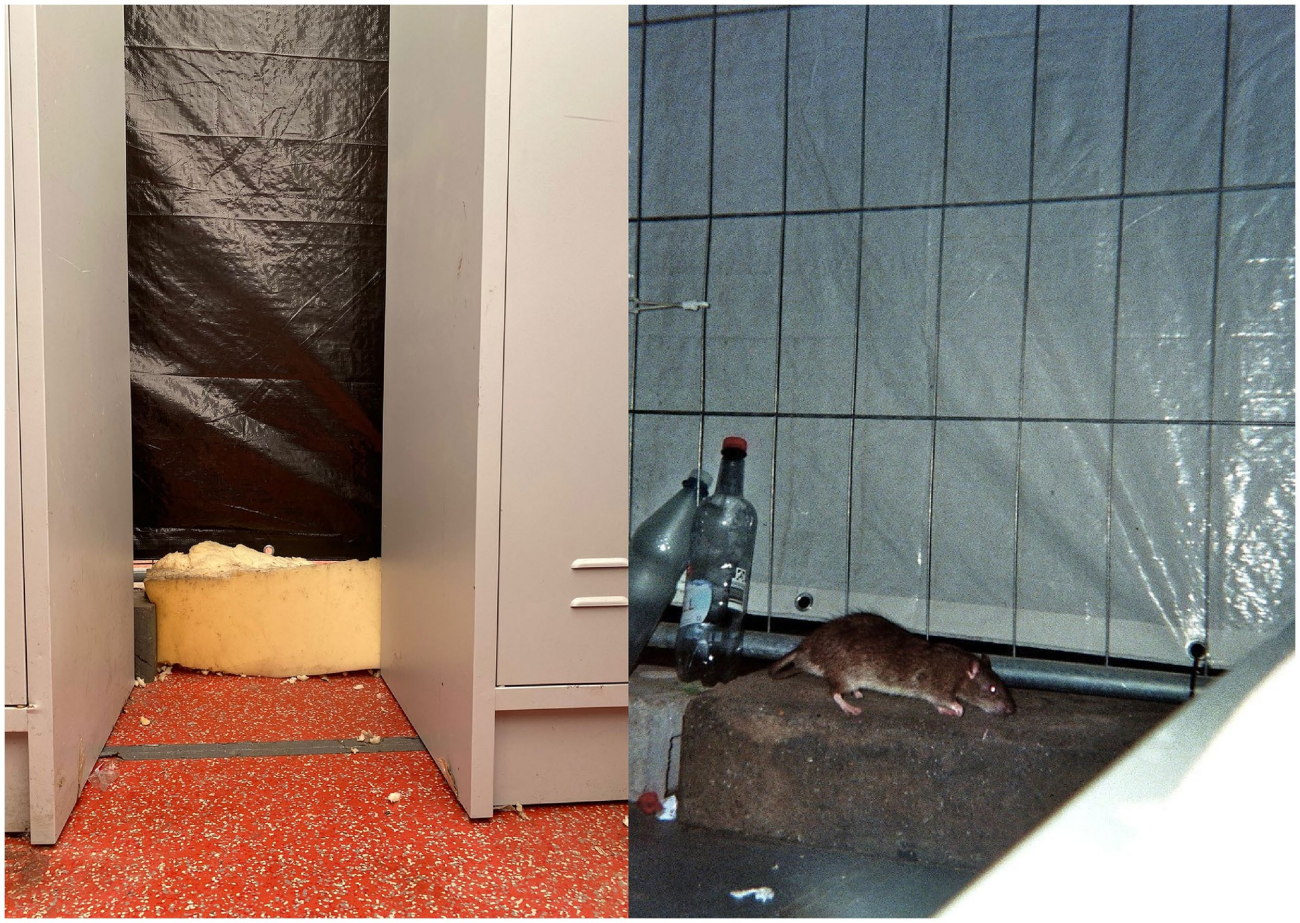
Left: Someone placed a torn foam mattress beneath the construction fence to prevent rats from entering, photo-documented by Ba (2023). Right: A rat near Maria’s bunk bed at night, photographed by Maria (2023).

**Figure 12:**
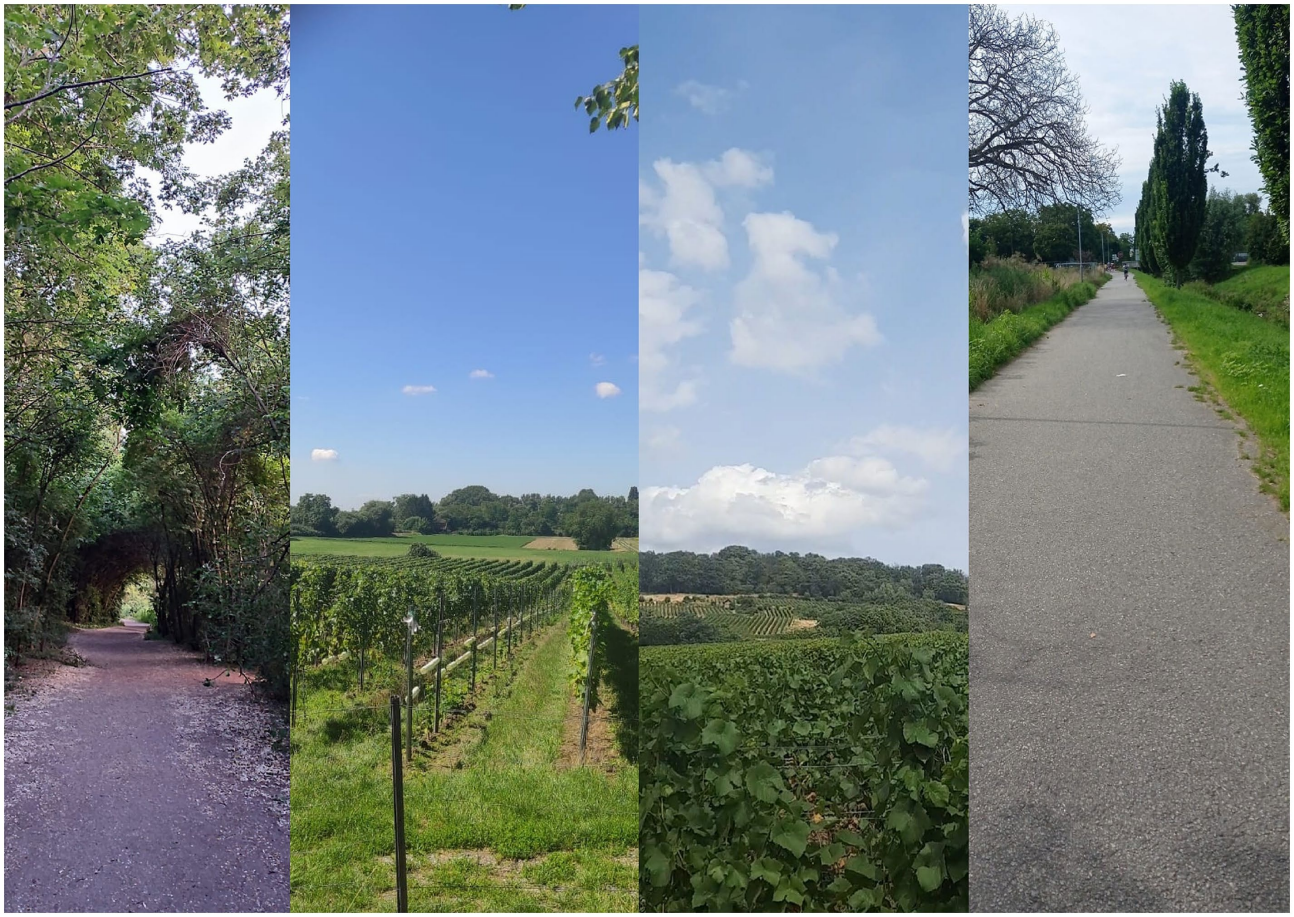
Walking on hiking trails near Bensheim, photographed by the respondents (2023).

**Figure 13:**
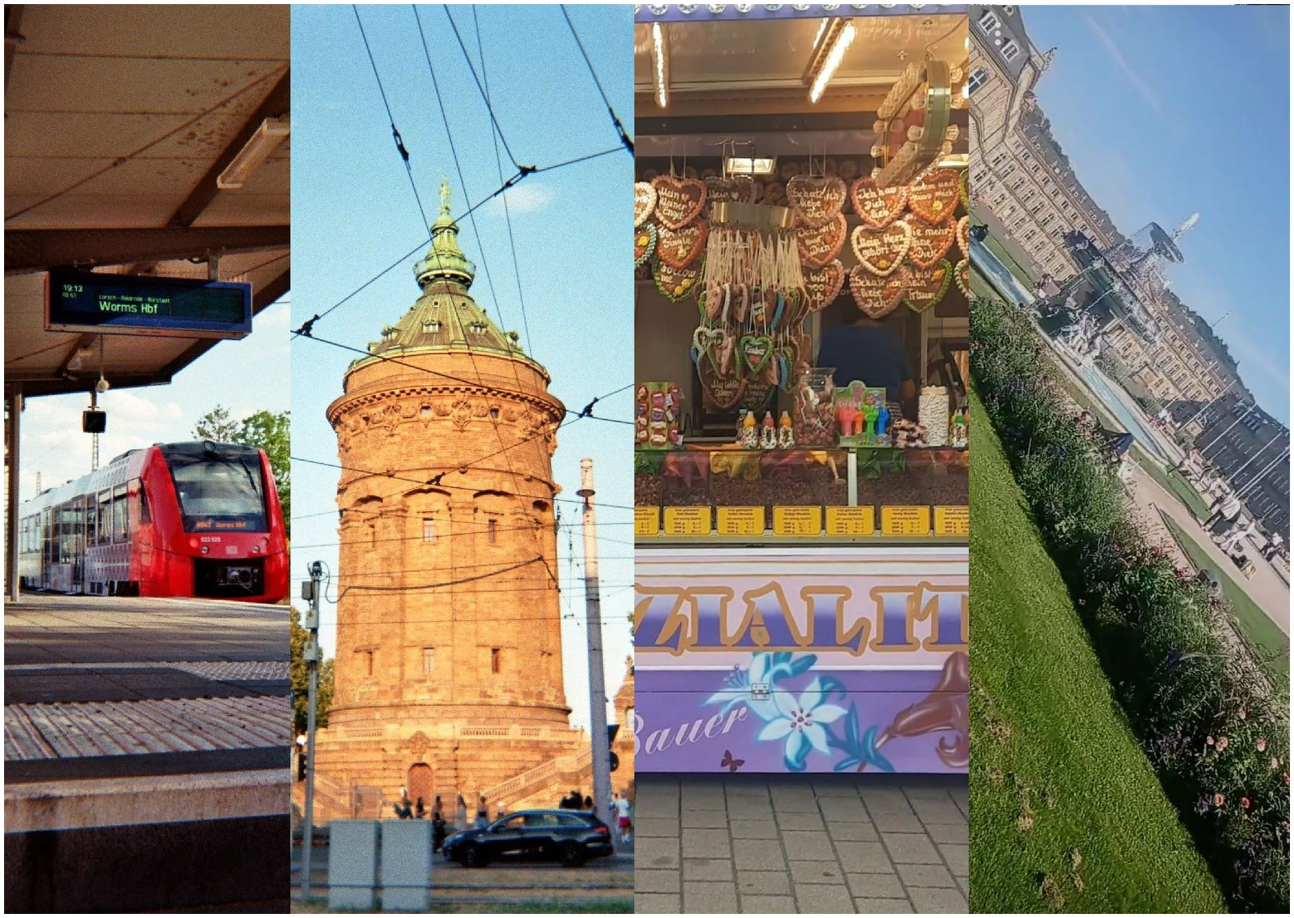
Visiting public spaces and sights with family and friends, photographed by the respondents (2023).

The question of the situatedness of photography is not only methodologically salient when asking for authorship and who has the right to photograph but also bears on who is eligible to interpret photographs and co-construct meaning. As den Van Scott (2018) shows, sociologists employ photographs in diverse ways—at times to illustrate their research findings (see Figure 1), but also to analyze and to find patterns throughout multiple sets of photographs (see Figures 2–13). Describing my stance on the use of photography begins by contextualizing my positionality, drawing on Bourdieu and Adamson (1990) concept of “epistemic reflexivity.” I do not focus on the “self,” as doing so risks reinforcing claims of authenticity (Bourdieu and Wacquant, 2022; Olmos-Vega et al., 2023; Wacquant, 1989). Rather, I aim to understand how applied methods embed “scientific-theoretical validation,” referring to an awareness of academic actions within the “organizational and cognitive structures of the discipline (trans. by CB)” (Bourdieu and Wacquant, 2022, p. 63–68).

Sociological inquiry traditionally prefers textual forms—such as interview transcripts and written academic outputs—over visualizations and photography. Bodies of text continue to dominate both the conduct and presentation of research. Where photography has been used in the past, its aims conflict with contemporary research ethics and epistemologies, because scholars used photographs illustratively or as “factual” academic findings rather than as situated knowledge (Richard and Lahman, 2015). Poole (2005) shows that photography employed in early anthropology was a “guilty pleasure” (p. 166)—initially used to depict race, then culture, then social organization—consistently aiming to erase the context in which anthropologists took images while disguising the ethnographers’ subjective effort as objective representation. Since the reflexive turn, scholarly interest in photographs has challenged traditional notions of objectivity (den Van Scott, 2018). Although from their discipline’s inception, visual ethnographers aimed to make social issues visible to a broader audience using researcher-generated photographs (Martiniello, 2017), for instance, of marginalized groups in their environmental settings (Harper, 2012), the academic debates are currently shaped by questions of engagement and levels of participation (Weber, 2019). This is specifically evident in relation to ethnographical approximation through photography in refugee research, where researchers do not want to expose respondents to any (re)traumatization (Tessitore, 2022; Weber, 2019). One proposal to circumvent such dangers when conducting research with refugees involves methodological reflection on ethics throughout the various levels of participatory research. The present study tries to meet these demands but is limited due to the experiential approach of the study.

As a white female sociologist from Germany and in my role as a seminar instructor on a “Spaces of Racism” unit at the Technical University of Darmstadt, my experiential approach to the research topic emerged from both an academic and pedagogical standpoint. I first became aware of the Bensheim tent city when master’s student Yasmin Eismayr expressed her interest in research under my supervision. Yasmin states that she positions herself as queer and is shaped by a biography marked both by upward mobility through academic education and broader familial trajectories of migration. She further characterizes her position within academia by a tension between the proximity to and distance from the institutional centers. These fields of tension significantly influenced the way she listened, asked questions, and co-conducted this research.

Visual ethnographic researchers and feminist scholars inform my understanding of photo-based methods. Pink (2008) emphasizes the importance of mobilizing visual ethnography to make sense of spaces. Rather than producing a “disembodied scientific objectivity,” I understand photographing as a medium of embodied scientific practice (Haraway, 1988, p. 576). At each stage, the photographic data, whether generated by researchers or respondents, serve as a tool (den Van Scott, 2018) to deepen my understanding, specifically geared to the various interplays of visualization and visuality of the spatial dimensions of racial discrimination.

## 2 Photo-based methods in the production of academic knowledge of refugees’ everyday experiences

In contrast to traditional, researcher-centered approaches that prioritize the researcher controlling data collection, handing participants the camera, and engaging in joint photo interpretation shifts priority, and perhaps authority, to their voices. In this section, I highlight both the benefits and disadvantages of making use of multi-layered photo-based methods in refugee research. Following the chronological outline of this research project, I begin with an approximation of the tent city through an aerial perspective. The next section deals with photo-documentation created during a “go-along”^7^ (Kusenbach, 2003) with a political representative, taken from our researchers’ or an outsider’s (etic) perspective. Finally, the respondents created photo-documentations, which we subsequently analyzed collaboratively (thus including an emic perspective). This last method is often referred to as “auto-driven photo-elicitation” and is otherwise known as “photo-interview.” We asked the refugees to photograph their everyday practices in and outside the tent city with disposable cameras. We then individually discussed a selection of their photographs with them to further clarify their perspectives. (See section 2.3 for a discussion of how I selected the photographs and presented them for discussion.) As I frame photographing as an embodied practice, both the *locale* of *extreme marginalization* and the epistemological framing of varying types of photographs play into this process of analysis.

### 2.1 Showing aerial photographs

We often use photographs to set the scene and explain our research to others (den Van Scott, 2018). Aerial photographs are central to these displays but are also cartographic practices that shape our everyday perceptions and actions (Gentzel and Wimmer, 2025). I argue that they also serve as a vantage point for academic knowledge production. Yet no other type of image is as frequently perceived as neutral and objective as the aerial photograph (Farman, 2010). Cartographic representations aim to reduce ambiguity and visually reinforce knowledge about territorializing structures. Readings of aerial photographs, as embedded in maps, are thus co-constitutive of the production of space (Lefebvre, 1991). Farman (2010) associates maps with machinic systems of representation, devoid of any human agency involved in capturing the image or compiling the data. I argue that gazing at maps is not merely a preliminary act of information gathering prior to entering the “actual” field. Map images actively shape the academic knowledge we produce and outcomes about the places we intend to study.

The choice of visual representation deeply entangles researchers in ontological relationality, mediating our interpretations of the Earth’s surface through mapping practices and the use of technical artifacts (Gentzel and Wimmer, 2025). “Maps, instead of being an objective visualization of a territory, are instead unstable signifiers, heavily imbued with the cultural perspectives of the society that created them” (Farman, 2010, p. 876). Mapping practices intertwine cultural expressions with imperialist knowledge production (Farman, 2010) and shape contemporary political processes such as the renaming and branding of territories and borders (Goodfriend, 2021). As feminist theorist Haraway (1988, p. 581) argues, the disembodied gaze shapes all visual technologies—a gaze that is inherently male, racist, militaristic, and hostile. On the other hand, the level of participatory engagement involved with maps critically shapes the outcome of the interpretation and implication of spatial representations. Farman (2010) claims that Google Maps allows mapping practices as a distinct program of participatory engagement. He argues that through engagement with Google Maps, users become embodied actors. Therefore, it is essential to distinguish between user-controlled forms of engagement and other, non-participatory mapping practices (Brantner, 2018). Either way, it is always the case that “by accepting the map as reality, the viewer enters into partnership with the map’s author over the hegemonic assumptions such a visual representation makes” (Farman, 2010, p. 878–879). Thus, any reading of aerial perspectives must first acknowledge the inherent partiality of geographical depictions. This acknowledgment challenges notions of territory depicted from their orbital vantage point. Within such readings lies the potential to unravel the presumed objectivity inscribed in aerial perspectives of refugee encampments.

### 2.2 Photo-documenting a go-along with a politician and humanitarian staff

Our photo-documentation functioned as a tool to foster an experiential understanding during a go-along involving the sociomaterial environment, the involved actors, and their situated interpretations. Lee and Ingold (2006) suggest making sense of places through walking, as individuals guide researchers through their everyday practices, unraveling the unconscious appropriations of spatial realities (De Certeau, 1985). Our ambition for photographing while walking was to transcend its conventional role as mere illustration in sociological research, instead serving to reflexively address the co-constitution of our research findings (Harper, 2012; Pink, 2001; Richard and Lahman, 2015; Schwartz, 1989). The go-along enables the documentation of spatial attributions while moving. It has proven particularly effective when combined with researcher-generated photo-documentation (Kusenbach, 2003; Richard and Lahman, 2015; Sommer and Töppel, 2021). We took 30 photographs while the political representative Matthias Schimpf and humanitarian staff explained the spatial arrangements and specificities of the tent city. Consequently, the images follow a sequential logic presented by the political representative guiding our tour—first, the entrance, then the “social tent,” the eating area, and the sleeping tents. Photographing while walking helped us explore the unfolding effects this place had on our perception (Pink, 2008). Even with the best intentions to “empathetically imagine ourselves into the places occupied” (Pink, 2008, p. 6), we must acknowledge that our sensory and emotional experiences inevitably differ from those accommodated in the camp. During the go-along, we became aware of restrictions and boundaries imposed by ethical considerations, such as avoiding trespassing, refraining from looking into prohibited areas, and not photographing people. We experienced the materialities through photographing in shaping and constraining our bodily practices.

To support our analysis, we made extensive field notes to complement the photographs, which enabled us to later revisit and critically reflect on our experiential understanding of the space. These notes include observations on our guiding professionals, sensory impressions, and atmospheric moments—such as feelings of attunement or disgust evoked by spatial configurations, marking personally perceived *spatial differentiations*. As we took our photographs from a stationary, upright, and relatively distant position toward the accommodated refugees, these omit a certain esthetic. Our photo-documentation largely lacks the presence of people, circumventing our stated objective to learn about everyday practices within the camp. Brief moments of eye contact with refugees, from a considerable distance, reinforced our sense of detachment. Photographing helped us to memorize sensed material aspects: heated tarpaulins attached to construction fences, scratched linoleum floors, used plastic chairs, and the rough surface of particle boards. For analytical purposes, our images serve as illustrative complements to the contextual and interpretive depth provided by our field notes (Figures 2, 3, 6, 7).

### 2.3 Analyzing auto-driven photo-elicitation with encamped refugees

We understand both the refugees’ self-produced photo-documentation and the joint analysis as a situated and participatory method (Pauwels, 2015; Prieto-Blanco, 2021). It foregrounds the agency of marginalized interviewees by positioning them as “‘knowledgeable’ informants,” rather than objects of inquiry (Pauwels, 2015, p. 98). We were motivated by the potential of having both verbal and photographic representations of “significant patterns of the respondent’s culture (norms, values, expectations, etc.) [that] can be expressed in the images that respondents make (both in what they depict and how things are depicted)” (Pauwels, 2015, p. 102). First, we visited the respondents individually in the tent city to communicate the methods, objectives, and ethics of the research. Based on their written consent, we conducted five semi-structured interviews in German that were simultaneously translated into the respondents’ spoken language by GRC staffers,^8^ focusing on the respondents’ biographical backgrounds, their experiences of everyday life in the tent city, and their feelings of alienation as well as perceptions of the surrounding urban and natural environment. Designing the interviews, we deliberately refrained from using terms such as “race” or “racial discrimination” to avoid framing their responses. Instead, we left the interpretation of potentially discriminatory experiences entirely to the respondents themselves.

With the subsequent joint photo analysis, we aimed to gain insight into the respondents’ subjective interpretations of the camp’s spatial organization as experienced through their everyday practices, as well as their perceptions of the broader urban–rural environment of Bensheim. We left it to the respondents to decide which of their photographs, and to what extent, they wished to refer to and use to illustrate perceived inequalities, experiences of alienation or discrimination, as well as stereotypical attributions toward them or made by themselves. We chose this approach to minimize the risk of causing further harm or retraumatization. The joint photo-elicitation did not follow a pre-scripted interview format. We only intervened with clarifying questions when respondents’ statements required further contextual understanding. We gave ethical considerations greater priority by letting the respondents decide which photographs they wanted to speak about rather than systematically addressing each image (Salma and Temuri, 2024). As we avoided introducing the issue of whether and how they perceive racial discrimination, this choice also introduced a conceptual limitation: a noticeable gap emerged between my initial curiosity about spatial manifestations of racial discrimination, the content of the images, and the actual narratives provided by the respondents about their everyday lives.

I want to further address several method-related limitations. We were forced to work around language barriers−neither of us spoke Ukrainian, Arabic, Turkish, or Farsi−and our limited knowledge of the respondents’ cultural contexts. To address this, the German Red Cross (GRC) staff supported our endeavor, helped with recruiting respondents, and provided simultaneous translation during the interviews.^9^ This setup between researchers, humanitarian workers, and refugees gives rise to several power imbalances. While we ensured confidentiality and clarified the independent nature of our research, the respondents’ relationship with the GRC staff remained a critical factor in interpreting the data. The refugees consented to participate after consultation with the social workers, with whom they were friendly or at least familiar. This raised further questions for us: how would their relationship with the present GRC staff affect their statements – would they fear criticism or even persecution? Given the ongoing institutional dependence of the respondents on GRC staff for essential services, we acknowledge that caution and strategic silences shaped their responses. Also, regarding simultaneous translation, how could we meaningfully interpret the respondents’ use of photography, and what might their visual choices reveal about their norms, values, and lived realities? In addition, I did not initially recognize the interviews as interracial (Marcucci, 2024), a recognition that would have been beneficial for researching racial discrimination (Wojnicka and Nowicka, 2024). In doing so, I unconsciously positioned myself as external to the research. I was arguably less aware of my own racial identity and the dynamics and power imbalances this implied. According to Marcucci (2024), we must address race or racism in interracial interview situations because in these contexts, relevant language is coded and requires sensitive interpretation. While anticipating all these limitations, I gave greater significance to the presumed advantages of the visual method, as it “evoke[s] emotional responses and alleviate[s] interview fatigue, researcher-participant miscommunication, and [even] lack of shared backgrounds” (Richard and Lahman, 2015, p. 6).

Women remained invisible in the accounts gathered during the go-along. However, studies suggest that women are particularly affected by discrimination and face specific challenges within refugee camps (Lenette and Boddy, 2013). Therefore, our sample for the joint photo-elicitation predominantly focused on female respondents. With the assistance of GRC social worker Alaa, contact was established with four women and one man residing in the tent city. Our sample size was limited to five participants for practical reasons. All respondents took part voluntarily. The duration of our data collection was confined to the summer semester of 2023. For these reasons, the sample is not representative.

The respondents took photographs using disposable cameras, each with a maximum of 26 exposures. We chose this approach to avoid requiring respondents to use their personal mobile phones. We asked them to photograph their everyday practices and living conditions in the tent city and Bensheim with no restrictions on locations, timing, or directives on what everyday life means to them. We then used their photographs as stimuli in a follow-up joint photo analysis (Pauwels, 2015). To choose the stimuli, I arranged the photographs chronologically in an analysis chart, where I added descriptive captions and assigned provisional categories. Descriptions were threefold: first, a non-interpretational description of the photograph was written. Second, categories were formed based on a “systematic and reflexive search for patterns” (den Van Scott, 2018, p. 724) that emerged both within individual photo series and across the full set of visual materials produced by all respondents, such as “nature photography,” “sleeping condition,” “activities outside the tent city,” and so on. From each individual catalog, I selected photographs for a joint photo-analysis (Richard and Lahman, 2015). The following criteria influenced the selection: the photographs depicted a range of dissimilar categories, displayed a distinctive visual composition, and depicted everyday practices. In contrast, I excluded photographs from the selection showing individuals, family members, or similar references. Finally, we came together for an open interview guided by their choice of referencing their preselected photographs. The duration of interviews ranged from 30 to 90 min, depending on the respondents’ level of engagement. We compensated the respondents in this three-stage study with €50.

Maria^10^ fled Ukraine in 2022 via Poland, Berlin, and Gießen with both her children. Formerly employed in higher education, she later worked in a “mini job.”^11^ At that time, Ukrainian citizens had access to work permits and social welfare money (*Bürgergeld*). In contrast, asylum seekers from “third” countries received considerably less social allowance (*Sozialgeld*), and the German state required them to apply for a work permit. In the summer of 2023, Maria had lived for two and a half months in the tent city. Supported by the GRC, she was soon to relocate to private housing, enabling her to speak candidly about the unsanitary and hostile living conditions in the tent city. She was highly motivated and aimed at “documenting the conditions under which the people live.” She took 26 analog photographs, plus 84 digital photographs, which she considered as backups.^12^ The main themes I identified in her photographs were nature and flowers, trips with her children, documentary-style images from inside the tent city, and artwork she created with the camp’s children on a voluntary basis.

Amina was in her twenties when she fled Syria. She quit her university studies there and was unable to attend classes after arriving in Germany. She had already resided in the tent city for 7 months while awaiting a decision on her asylum case. She underwent treatment for depression and malnutrition. While she, too, voiced strong criticism of the poor living conditions, her narrative was more desperate, rooted in her limited mobility due to her health condition, which prevented her from walking long distances. She produced a total of 20 analog photographs, primarily capturing scenes from within the tent city, as well as images of nearby sites in Bensheim and public transportation.

Omar, a man from Syria, was in his 40 and had been in the tent city for 1 month. He formerly worked in transportation but currently has no other means of transport than to walk, sometimes as far as to the next town. He also suffered from poor food conditions. Although most of the refugees were other Syrian men, he stated that he encountered multiple barriers to engaging with most of them. He took 12 analog and four digital photographs. The main categories include nature and hiking trails, the home of a Syrian friend, and cityscapes of Bensheim. His perspective revealed several comparable points to the experiences of female refugees.

Sara was in her 40 when she fled Iran, leaving a teenage child behind−a fact she initially withheld in the interview with us, as was pointed out by the accompanying social worker. She used to work as a project coordinator and had friends in southern Germany, whom she visited frequently. She also used the interview to complain to the social workers about unhygienic conditions and an acute case of scabies. Her catalog consists of 20 analog photographs, all depicting either tourist sights around Bensheim or landmarks in cities in southern Germany.

Khadija from Afghanistan was in her 20s, had lived in the tent city for 4 months, and was experiencing severe health issues while waiting for a decision on her asylum case. She mainly socialized with other Afghan families and came across as reserved and worried. Her catalog consists of eleven analog photographs, all showing images of the lake in Bensheim taken from a 45-degree angle, as well as sights in Mannheim.

Reading images—especially photographs produced by refugees—presents specific challenges, such as the necessity to deepen our understanding of the respondents’ cultural backgrounds and decode the meanings embedded in their images (Pauwels, 2015). Socioeconomic, national, or cultural proximity between photographer and interviewer surfaces more information, as it creates a higher level of trust (Kappelhof and De Leeuw, 2019; Marcucci, 2024). In hindsight, I recognize that this was the case with Amina from Syria and especially with Maria from Ukraine. As suggested by Prieto-Blanco (2021), despite the language barriers, I felt greater proximity toward them, since they both held academic degrees and belonged to a comparable social class. Moreover, Maria not only produced more photographs than other respondents but also invested more time in the project, enabling deeper collaboration and offering a greater understanding of her motives and interpretations. This highlights how joint photo-analysis requires an elevated level of cooperation—not only to produce visual material but also to engage in its contextualization. In contrast, the photographs taken by Khadija, Omar, and Sara—all of whom were still awaiting decisions on their asylum status—exclusively depict places outside the tent city that they visited in their free time. None of their images showed the interior of the camp. While they spoke openly about the difficulties of their living conditions, they chose not to photograph them. This gives rise to at least two methodological reflections. First, the power asymmetry between the respondent and researcher: the respondents might have perceived us as complicit with the German institutions they depended on. Second, photographing served the respondents as a strategy of relief or deflection from daily challenges. They deliberately avoided documenting spaces of dehumanization, instead focusing on expressions of personal agency, such as walking paths, lakes, or natural scenery.

## 3 Spatial dimensions of racial discrimination from aerial, etic, and emic perspectives

### 3.1 Aerial: showing the periphery

*What do I perceive when I see the tent city of Bensheim from an aerial perspective? (*Figure 1*). Between green fields and small patches of forest, rows of neatly planted trees, sports fields, the shimmering turquoise of a bathing lake, the gray lines of intersecting roads, roundabouts, and the nearby highway cutting through the landscape, separated by a belt of large perimeter block buildings of the smaller single-family houses with red, gray, and brown shingles and tiny gardens, lies a large sandy area with four equally sized rectangular tents. For a moment, my eye rests on these precisely arranged rectangles, which inscribe flatness into the social texture of the place—the uniform regularity of the camp, in beige and white, representing an anonymous mass of inhabitants.* (Field note, 10 May 2023).

Presenting a map of the tent city from an aerial perspective produces a mere spatial representation that gestures toward the marginalization of refugees residing at Bensheim’s periphery. Without a critical engagement with the ideological framing embedded in the medium itself, the aerial view risks reproducing a distanced, voyeuristic gaze (Farman, 2010). As Santos (2018) argues, this cartographic form scales and restructures the spatial reading—one that renders space manageable and maneuverable, thereby abstracting and depoliticizing the lived realities it purports to represent. Critically engaging with the map as a purposeful spatial technology of governance, I further unpack the context of contemporary refugee movements in Europe. Drawing on Balibar’s (2004) work on borders, a rereading of the aerial perspective discloses that we have entered an era marked by the dispersion of border (land)s within Europe. Similarly, Stehle (2006) shows how the EU internalizes border control to inner zones. Through this visual representation, I understood the tent city as a material manifestation of *spatial dissimilation—*portraying the shift toward the reconfiguration of borders and the regulation of mobility within the confines of German municipalities.

Situated within the territorial boundaries of Germany, yet characterized by multiple disadvantages from outside German society, the tent city emerges as a categorized, peripheral site where we assume that surveillance takes place. In this context, I interpret the aerial photograph as an “enabling technology” (Delaney, 2002, p. 7) that visually (re) produces and materializes racial differentiations. As Delaney puts it:

This argument suggests that the territorial division of continuous social space into dichotomous “insides” and “outsides” facilitates the polarization of a continuous range of colors (browns, beiges, tans, and pinks) into “white” and “black” and hence the freezing of identities into “we” and “they” (Delaney, 2002, p. 7).

Here, the photographic representation (Figure 1) serves as a hegemonic tool. As such, it reinforces the sociospatial and symbolic segregation of refugees from the host society. This constructed binary is intricately bound to a national biopolitical regime, which I refer to as a *spatially enacted classificatory system*. The aerial photograph functions as a representation of space—an image shaped by regimes of control. This representation portrays the multifaceted disempowerment of the inhabitants—rendering them *homines sacri*, stripped of any legal recognition—and positions the tent city as a paradigmatic “space of exception” (Agamben, 1998; Grbac, 2013; Stehle, 2006). The visual representation entangles the term “refugee” with territorial imaginaries embedded in the “birth-nation nexus”—a framework that distributes and conditions different rights to citizenship (Agamben, 1998, p. 131–132).

In my reading, the aerial perspective not only abstracts space but also obscures everyday practices, appropriations, and spatial interpretations enacted by its temporary inhabitants—thereby disempowering them twice over. First, it renders them, through a logic of oversight, as an anonymous and de-individualized mass. Second, it erases the political and social agency embedded in their presence. Echoing De Certeau (1985, p. 124) we must descend to eye level to truly perceive the tent city and understand how “its practitioners employ space.” In interpreting the aerial photograph of the tent city, I reframe it by rereading it as a map, a tool of government—one that inherently (re)produces entangled logics of biopolitics and citizenship.

### 3.2 Etic: researchers’ perspective on architectures of forced departure

*The heat in mid-June almost becomes unbearable as we wait at the entrance to the tent city to conduct our go-along with district representative Matthias Schimpf. The glittering surface of the fenced-off lake across the way comes into view—appearing inaccessible from where we stand. We decide to walk along the access road and observe that the management enclosed the grounds with double wire mesh fences anchored in concrete bases, constructed from durable wire rods−a technique commonly employed for construction-site security and within the industrial sector (*Figure 4*). We come across a narrow footpath winding through a wooded area, leading to the gas station adjacent to the highway. Passersby left the well-trodden path littered with cigarette butts and discarded bottles. We discuss that these objects bear witness to signs of the few accessible outlets for consumption, but also of neglect, beyond the confines of the camp.* (Field note, 26 May 2023).

As we pass through the entry checkpoint built in a container, monitored by security personnel, using fingerprint and card scanners (Figure 5), we sense a juridically differently framed space, highly surveilled and restricted in terms of both access and leaving. One of the GRC workers, Alaa, agreed to accompany us on our walk with Mr. Schimpf. Alaa fled Syria in 2015 and first registered in Bensheim, which subsequently meant that he had to stay there.^13^ His experience as an individual with a refugee background who also occupies an institutional role within the German Red Cross challenges conventional narratives of victimhood. His dual positioning invites a reconsideration of agency, particularly in the context of framing Germany as a “post-migrant society,” as argued by El-Tayeb (2016, p. 20). She contends that by critically reassessing how Germany constructs its historical narratives, we can better recognize and validate the diversity that characterizes both past and contemporary German society. In this light, Alaa challenges preconceived notions of refugees in camps—such as passivity, dependence, and hopelessness—by defying expectations of what a person might do in that *locale*.

While the district representative shared information about the camp’s demographics, we observed a few men scattered across the area at a considerable distance. Nothing conveyed the impression that the site currently accommodated 400 refugees (Figure 2). Matthias Schimpf explained that today’s male inhabitants from Afghanistan belonged to a second “wave of refugees,” a term commonly used in German discourse about refugees to express the overwhelming distress of the host society, evoking images of natural disaster. “These” men were socially distinct from earlier, wealthier arrivals. They were without formal education and had made it through immense hardship—some walked over 3,000 kilometers, and some paid smugglers. Nevertheless, the majority of “them” had no prospect of securing asylum in Germany.

This vignette illustrates (Hirschauer, 2023, p. 86) observation that “first, in egocentric we/they distinctions, the two sides are unequally de-differentiated: the own group tends to be perceived with greater internal differentiation, based on a narcissistic and homophilic investment in the ingroup, whereas the other is more strongly homogenized and essentialized” (trans. by CB). Echoing this, Mr. Schimpf frequently joked during the go-along with male staffers in key institutional roles, recalling problematic encounters involving “young, unaccompanied, single men” from “Arab countries,” whom he identified as the primary “source of trouble.” This form of othering reveals the essentialist framing of this group, marked by implicit hierarchies of superiority and inferiority (Hirschauer, 2023). As we moved through the tent city, we noticed that this ideation is inscribed materially into the camp’s spatial organization: a top-down segregation scheme emerges through zones of surveillance, such as the monitored public space, and separated containers designated for security personnel and GRC social workers. Entering the social tent, it becomes evident that control was also sociospatially enacted through the time-restricted distribution of meals and curfew times, as well as by fencing off spaces, leading to embodied practices of waiting for the inhabitants (Figure 6). In the corner of the social tent, a few young men sat quietly with their backs turned to us, barely acknowledging our presence. Pointing toward them, Mr. Schimpf further explained that the power strips in the tent were the only possibility for the inhabitants to charge their devices (Figure 7).

The go-along and photo-documentation, as an embodied act of distancing from “these men,” led to our deduction that the tent city was an architectural objectification of EU regulations, a *spatially enacted classificatory system*. The overall spatial constitution links to the following classificatory binary—nationals from “Islamic/Muslim” cultures are framed as backward in contrast to the ideal of the “European Enlightenment” (El-Tayeb, 2011). These mechanisms not only systematically devalue associated groups on multiple levels but also reflect broader patterns of structural exclusion embedded within European migration regimes (Stehle, 2006). Through the externalization of this classificatory logic, the built environment visually and materially expresses what Wacquant (2007) describes as the perpetuation of “territorial stigmatization,” ultimately affecting all inhabitants of the camp in their lives within and beyond the camp.

*We continue our go-along in one of the inhabited tents. Upon entering, we observe rows of construction fences draped with tarpaulins and bed sheets (*Figure *3**). Fans rotate, though they barely seem to alleviate the stifling heat. A young woman, with a toddler at her feet, looks at us expectantly when she sees us entering with Alaa, the social worker. Opposite her, a man steps out of his compartment by lifting a bed sheet, which Alaa tells us serves as a door. Alaa explains the domiciliary rights, stating that we may only enter the compartments with the permission of the residents or in cases of imminent danger. Otherwise, anyone’s entry is a violation of the “home.” We leave the tent city without looking into one of the individual compartments. Although the process of photo-documentation gives us a bodily sense of the spatial divisions, restrictions, and precarious living conditions, no moment is more striking than leaving—the simple act of walking away into the midday heat makes the sociospatial inequalities most palpable*. (Field note, 26 May 2023).

Drawing on our photo-documentation and the ethnographic field notes of the go-along, we summarized the following: The tent city primarily functions to spatially enforce a distinct interplay of idealized classificatory concepts, such as gendered, social, stereotypical, ethical, and racial categories. At this point, our photo-documentation informed our understanding of the spatial dimensions of racial discrimination as a scheme of *territorial stigmatization* (Wacquant, 2007), primarily reproducing culturalist and essentialized characterizations of “young Muslim men”^14^ and incorporating recurring tropes of Islamophobia (Attia, 2012; Sadeghi, 2019). The management specifically designed the spatial aspects of confinement based on a conceptualization and legal categorization of this collectivized group. I therefore call the tent city not only a site of “forced arrival” (Kreichauf, 2018), but also an architecture of “forced departure”—designed solely to detain men temporarily and under the lowest possible living standards. Internally, the lives of refugees are under spatial constraints, which eliminate privacy and spaces for retreat from camp life and sustain a surveilled space by an overwhelming presence of men, both residents and staff. Walking with a politician and humanitarian staff, however, the one-dimensional image of “the refugees” imposed by the aerial photographs began to unravel. We did not find ourselves confronted with an anonymous mass but with several—mostly male—actors possessing differing forms of agency and situated within social hierarchies shaped by class, national, and ethnic differentiations. Within this space, ideated to contain “young Muslim men,” we concluded that women remained entirely invisible and silent.

### 3.3 Emic: insider perspectives and the multivocality of spatial dimensions of racial discrimination

As Malkki (2002, p. 358) notes, “there is no such thing as a typical refugee experience [nor] a typical refugee camp.” Photographs produced by encamped refugees and their joint analysis prove to be a valuable tool for gaining insight into diverse and overlapping spatial dimensions of racial discrimination. All respondents suffered under the cramped and shared living conditions, which fostered various forms of human categorization—including stereotyping, generalizing, and mutual attribution—among the refugees themselves (Dizdar et al., 2021; Hirschauer, 2021). The consequences of this *spatially enacted classificatory system* of imposed asylum policy affected all respondents—a phenomenon framed in academic discourse as “institutional racism” (Kreichauf, 2018). However, the joint photo analysis contained little or no explicit criticism of the German host society or the European Union, contrary to what we had initially assumed based on the analysis of the go-along. This absence challenged our assumptions and revealed a disconnect between what we, as researchers, sought to find about racial discrimination and what the respondents chose to show or articulate about their everyday lives. I suggest explaining this gap both through the respondents’ uncertainty surrounding their legal status and the potential fear of expressing critical views, and by the inherent constraints of photographic representation. Moreover, the differing conditions of waiting and the unpredictability of their length of stay in the tent city likely shaped how respondents engaged with and inhabited the space, influencing what they depicted or deliberately left out in their photographs. We noticed that even the most intimate and protected space—the compartment—creates physical and psychological stress. Sharing compartments within a large communal tent intensifies existing stereotypes and prejudices by female residents, particularly toward men and those perceived as belonging to “other” nationalities and ethnicities. The forced accommodation in a multi-ethnic encampment does not yield empathy for other refugees but surfaces manifold notions of human differentiation. The respondents did not verbally attribute or share common notions of race or racism. They drew their differentiations from differently perceived notions of “culture,” “nationhood,” or “ethnicity.” I argue that we need further investigations into how tent-based camps, as *spaces of extreme marginalization,* reinforce human categorization.

#### 3.3.1 Gendered relations: ubiquitous and unsettling male gazes

One of the main stressors was that the tent city functions as a space of containment and control, organized through zoning, and described by Khadija as “prison-like conditions.” Feelings of unease stemmed not only from a general sense of surveillance by male security staff but also from the presence of male co-inhabitants. Among the most frequently mentioned forms of surveillance was the system of food distribution, which, for respondents, acted as a form of biopolitics: it restricted their ability to prepare and eat food when, how, and with whom they wished. All the respondents stated that the food was of the lowest quality and damaged their well-being. Maria recalled that some inhabitants “pay money in nearby hotels so that they can prepare a meal.” In contrast to their verbal accounts about the conditions of nutrition, none of the respondents depicted the food distribution area in their photography. However, all produced counter-images from taking meals outside the camp—featuring items such as ice cream, pizza, lemonade, or wine. Getting food and beverages outside meant escaping the camp confines and living like a person with agency and identity.

However, other forms of surveillance within the camp were presented. During her photo-elicitation, Amina first revealed her fear that the camp management surveilled her bed.

*In her photograph, Amina points toward a black rectangular mechanical device mounted on a pole, attached to a wire (*Figure 8*). “I am being constantly watched,” she adds to her caption. Alaa, the social worker, leans over and, astonished, explains that it is a thermostatic device used to measure humidity and temperature inside the tent. She looks back at the picture and adds, “And I thought I was being constantly filmed in my bed.”* (Field note, 18 August 2023).

Although her photograph did not focus specifically on the horrifying discovery she thought she had made, this vignette perfectly shows her perception of being surveilled by a voyeuristic Other. All female respondents stated that men used the dense conditions of the tent city to control and surveil women. Sara expressed deep frustration over her unsuccessful efforts to initiate a women-only tent, hoping to avoid stalking, unsolicited advances, and the disturbing presence of men misusing alcohol and drugs at night. The camp management denied her request due to infrastructural and financial constraints. Maria experienced how both male inhabitants and security staff did not add to an overall feeling of protection but of controlling her as a woman: “There are so many lonely men who are hungry. There was one man following me everywhere. In this case, the security guard told me to just ignore him, but [the man] was later removed from our camp to another one.” Gendered discrimination also becomes evident in their accounts of exerting control over their use of space. Amina, for instance, highlighted her discomfort using the shared social tent to charge her mobile phone, as young men consistently occupied the space, making her feel vulnerable. Accordingly, she took no photograph of the social tent. Khadija, adhering to strict religious norms, also avoided any male presence and preferred to visit other Afghan families with children at a nearby playground—“but [only] if there are no refugees who sit and drink alcohol, of course. Because many Ukrainians drink alcohol there, but only in Ukraine.” Her statement underscored not only the gendered spatial dynamics but also revealed her own stereotypical assumptions, because she felt her ability to practice her Islamic religious and cultural values was hindered by physically distancing herself from men. Moreover, her statement indicated a self-positioning that distinguished her from the “refugee” category, exempting herself from this classificatory system.

#### 3.3.2 Marginalized others: shared spaces as conflict zones

*Maria runs all the printed photographs through her hands. Occasionally, she pauses and sets one aside. Then she places the stack on the table and points to the image she took of the toilets. The image shows the inside of the toilet container: someone tore down the shower curtain and ripped off a showerhead. Dirt and water accumulate at the bottom of the shower tray, forming a dark streak (*Figure *9**). She attributes the depicted unsanitary conditions to other female residents, stating that this was “caused by women of other nationalities, because different nationalities pay different attention to cleanliness.”* (Field note, 28 August 2023).

The photographs and statements of unsanitary conditions in shared female spaces reveal how female respondents reproduce racialized and ethnicized stereotypes—serving as a mechanism to establish moral hierarchies and to distance themselves from other marginalized groups. While Maria left unspecified which groups she refers to—apart from distinguishing them from her own ingroup of “Ukrainians”—Amina explicitly attributes unhygienic usages of sanitary infrastructures to “gypsies,” a pejorative and racist term for Roma and Sinti. Hence, the shared use of sanitary facilities revealed discriminatory slurs and prompted avoidance strategies, such as showering early in the morning, soon after the cleaning staff left. Their narratives illustrate how they internalize social divisions, shaped by broader discourses of cultural superiority and cleanliness, and how they enact these in confined, high-pressure environments like the tent city. Importantly, their own photographs indicated not only perceived intergroup tension but also how they moralize structural neglect along ethnic lines, rather than recognizing and addressing their inadequate living condition as a systemic failure.

The photo-elicitation proved to be a sound method for investigating perceived misuse of sanitary infrastructure by others. However, the method fell short when women wanted to share a personal account of individual distress. For instance, Khadija, an Afghan woman suffering from endometriosis, only disclosed in verbal accounts what severe physical and emotional reactions she has toward these facilities, leaving no photographic evidence. She stated that the outdoor placement of toilets posed a significant barrier to use for her, especially during menstruation: “You cannot even look at the toilets. When I am having my period it is bad. I have no medication. I have no proper doctors. I have something in my ovaries, and I am really messed up. Yes, so I am looking for a gynecologist right now.” Her verbal testimony highlights how the lack of access to basic healthcare and shared sanitary infrastructure intersects with female health vulnerabilities in the tent city. However, photographing proved particularly challenging to document an absence, a void, or a lack of something. This was particularly difficult in relation to embodied experiences of individual suffering, which resist straightforward visual representation.

Staff members of the GRC assigned female refugees to compartments with multiple bunk beds, showing little regard for personal needs or interpersonal compatibility with co-residents. Allocation by GRC social workers was based on gender, then loosely on nationality, language, or age, but not on individual preferences or vulnerability. This arrangement did not automatically foster solidarity among women. Khadija recounted how difficult it was to share a unit with an Iranian woman who, despite being aware of her serious health condition, showed no signs of support or empathy. Amina photographed how she protects her belongings, keeping everything packed in her backpack during the day and carrying it with her (Figure 10, left). In contrast, Maria, a Ukrainian mother of two, felt fortunate as the GRC assigned her to a unit with another Ukrainian woman and her daughter. Yet even under these relatively favorable conditions, she described the psychological toll of constant vigilance. In one of her photographs, she emphasized how the low partitions made from construction fences failed to provide any real sense of separation or security from the upper bunks of neighboring compartments (Figure 10, right). As a coping strategy, she assigned the upper bed to her son and slept on the lower one with her daughter, trying to create a minimal sense of safety and intimacy in an otherwise exposed setting. Sleeping below also held moments of dehumanization, when Maria simply pointed to one of her photographs: “Rats.” She managed to photograph a rat at night next to her bed and explained how other inhabitants tore up unused foam mattresses and stuffed them underneath the construction fences to prevent rats from entering (Figure 11). Their description allows me to deduce how the imposed *spatial dissimilation* in a *space of forced departure*, designed for an imagined, homogenous group of “young Muslim men,” complicated and reified social, gendered, and ethnic categorizations among women.

#### 3.3.3 Escaping extreme marginalization: walking as a coping strategy

Out of their total photographs, Maria depicted natural landscapes and flowers in 49 of 100 images, Omar in 18 of 19, Amina in one of 20, and Sara in 19 of 25. Meanwhile, Khadija featured landscapes, nature, and hiking trails in six of her 11 images. The spatial design of the camp, which caused a lack of privacy and was compounded by tensions among other inhabitants, left only one viable form of adaptation: leaving as often and for as long as possible. “And when you are here, it depresses you. You must be outside as much as possible,” Maria explained. Amina similarly recalled her initial reaction upon arrival: “I was outside all the time. I was out walking for five to 6 h a day because I was not used to being here in the camp.” Her constant movement through Bensheim was both an act of avoidance and an attempt at self-regulation in opposition to the imposed *spatially enacted classificatory system* of being “a refugee.” However, the physical and psychological effects of this constant displacement became increasingly apparent. She began to lose significant amounts of weight, and as her strength declined, so did her capacity to maintain her daily walking routine. “I cannot walk as far anymore,” she told us. Consequently, all her photographs document the embodied effects of prolonged peripheralization and stigma—depicting deserted local playgrounds, clothing donation containers, and the train station of Bensheim. Through the spatial range captured in Amina’s images, the camp’s socio-spatial conditions erode not only the will to resist but also the physical capacity to do so.

All respondents photographed their central activity of walking the hiking trails and vineyards in the surrounding Bergstraße district (Figure 12). Both Omar and Sara stated that they usually went for walks alone. Consequently, one of the main patterns in photographs features empty landscapes and hiking trails around the tent city. Referencing their photographs, all stated that walking functions as a refusal, a coping strategy, and a subtle form of resistance against the classification as refugees and the pressure exerted through the implicit multi-layered forms of discrimination. As De Certeau (1985, p. 120) asserts, “walking is selecting”—a practice through which spatial order is both navigated and subverted, revealing and reproducing the underlying conditions embedded in the built environment. However, in this context, walking was far from playful or subversively liberating: it became a vital form of psychological survival. Simply going outside did not in itself constitute a process of intrinsic value or have a direct impact on the respondents’ psychological well-being. As Omar, Sara, and Khadija explained, meaningful encounters—meeting friends or people who shared the same language or nationality—were crucial. They took photographs from the passenger seat (Khadija), on the balcony of a friend’s apartment (Omar), during city visits and her mini job as a cleaner (Maria), and on walks with friends (Sara), stating that their well-being depended on nurturing social networks (Figure 13).

The photo-documentations provide unexpected accounts of experiences outside the tent city, revealing how their flight from spatial classification as “refugees” coincides with *territorial stigmatization*. Rather than taking photographs of their everyday practices within the confines of the tent city, the respondents chose—photographically and narratively—to highlight how their activities outside the camp serve as forms of coping and connection, offering them a sense of self and belonging. The intentional photographic absence of images depicting scenes, objects, and people from inside the tent city underscores this flight from the camp. When we asked Maria to comment on some of her photographs taken inside the tent city, she quickly swiped them away and said, “Now, I just want to forget.” Her rejection shows how she wants to be perceived—not as a passive recipient or subject of institutional control or classified “refugee,” but as an individual with agency, with friends and family members embedded in broader social networks. Stepping out of the tent city, in turn, meant reinserting oneself into a community—building bonds with others who shared the same nationality, age, gender, culture, or values.

## 4 Discussion—reflecting on intersecting perspectives on spaces of extreme marginalization

In this article, I have highlighted both the potential and limitations of various photo-based methods for researching spatial dimensions of racial discrimination, as well as the epistemology inherent to each type of photography. I discussed photo-based methods in refugee encampments as embodied practices of situated knowledge production (Haraway, 1988). In what follows, I will reflect on (1) our initial assumption that insider perspectives are to be favored above others and (2) the necessity of taking the sociomaterial environment into account when researching spatial dimensions of racial discrimination in refugee encampments.

First, I address the considerable gap between our literature-based assumptions about forms of racial discrimination, let alone their spatial dimensions, and the results of the assignment to photograph everyday life. Perspectives from both researchers and respondents validated the assumption that the tent city constitutes a “space of exception” within refugee housing in Germany and serves as an example of the EU-wide *spatially enacted classificatory system* imposed through the architecture of accommodation. While the go-along interview with the local politician offered no further insight than the *spatial dissimilation* of refugees and the host society, the photographic practices of refugees usefully addressed the making of gendered, ethnicized, and racialized dynamics within the refugee group. From them, I drew conclusions about the manifold and intersecting spatial dimensions of racial discrimination, which not only act on “the refugees” as a category but also highlight their own systems of categorizations ascribed through prejudices and stereotypes.

The different photographic practices of researchers and respondents functioned as a bodily seismograph, registering the spatially (re)structured power relations of the photographers in relation to the social space. I showed that the distinction between researcher- and respondent-produced photographs transcends a simple dichotomy or chronologically additive perspectives (cf. Pauwels, 2015). I argue that in this case, photo-documentations with different authorships unravel assumptions, bridge and relate diverse forms of knowledge production, or contest established interpretations on the spatial dimensions of racial discrimination. These interactions both challenge researchers’ preconceptions and contribute to a more nuanced and expanded methodological discussion, as I will show in the following.

I firstly established that the aerial perspective allowed the deduction that the camp was a “space of exception”—a space that reduced its temporary inhabitants to a collective mass, stripping them of any right beyond bare life, rendering them *homines sacri* (Agamben, 1998). Consequently, I assumed that mistreatment by the German host society and the camp’s living conditions would emerge as the primary driving factors in the respondents’ photographing spatial dimensions of racial discrimination in their everyday practices. Although the poor living standards in the tent city were a dominant narrative, “German” society was rarely, if ever, explicitly held responsible for what Khadija described as a “prison-like condition.” Due to our assignment to the respondents to photograph their everyday life, their photographs did “not include details central to questions of interest to the researcher” (Pauwels, 2015, p. 106) and avoided a straightforward reading of racial discrimination on that level. I hence frame the first conceptual assumption as that of “Agambian exceptionalism” (Bulley, 2014, p. 67; cf. Sanyal, 2012), which obscured the study’s proclaimed assumption to learn about the respondents’ critique of national or EU-wide legislation. This assumption of exceptionalism, however useful at first, failed to relate the local sociopolitical struggles to the subjectivities of refugees. Through our outsider perspective engaged during the photo-documented go-along, I deduced the presence of the inclusive/exclusionist *spatially enacted classificatory system,* acting through the ideation of an ethno-essentialist group of “young Muslim men” (cf. Attia, 2012) living within a built environment structured by supranational, national, and federal legislation. This insight revealed that the camp was overtly imagined by the camp management as a space of forced departure. The researcher produced photo-documentation showing the tent city’s function as a space of containment, of actively restricting “young Muslim men” from moving, and of their idealized departure by policy makers. Beyond this spatially reproduced ideation of “refugees” as “young Muslim men,” insider perspectives from female respondents revealed how this *spatially enacted classificatory system* played out negatively for them, as the management did not design the encampment for them. I learned from their perceptions on the specific sociomaterial manifestations of *territorial stigmatization* how it was dehumanizing for them, challenged them psychologically, and bodily restrained them. Their jointly elicited photographs revealed the threats to their physical and psychological well-being, such as the risk of exposure to men’s harassment. For them, national differentiations of men surfaced stereotypical categories [e.g., that (only) Ukrainian men drink], which led to strategies of avoiding shared public spaces. The respondents attributed issues of uncleanliness inside the camp to women of “other” cultures, ethnicities, and national backgrounds. Respondents clearly deployed racist anti-Roma/Sinti categorizations (“Gypsy”) in these shared spaces. I argue that the camp’s spatial organization reinforced existing prejudices and stereotypes among refugees and might even promote differentiation and categorization. This raises the need to investigate further whether photo-documentations produced by refugee respondents, when applied in contexts of *extreme marginalization*, facilitate the articulation of racist resentments and stereotypes.

In my understanding, this is precisely where the question of perspectives and *locale* intersect. In the tent city, the *locale* influences what refugees can or cannot photograph or visually represent. In a *spatially enacted classificatory system*, the absence of insider imagery or the refusal or inability to capture certain spatial experiences reveals more than, from a positivist stance, photographs do. These limitations can become productive, pointing to how refugees perceive and perform silences, erasures, and invisibilities that co-constitute spatial dimensions of racial discrimination.

The act of “giving people a voice” through joint photo-analysis in such an environment does not absolve researchers from critically reflecting on ethical constraints or their own racial situatedness that may reproduce inequalities and power asymmetries within the researcher-respondent relationship and presentation of research results (Harper, 2012). Future refugee research on racial discrimination using photo-based methods must acknowledge the housing conditions and ground the researchers’ understanding in the environment and the specific *locale* where refugees take photographs. Researcher- and respondent-conducted photo-documentations as embodied seismographs can enrich these place-based understandings and require researchers to immerse themselves in the *locale* and, during interpretation, actively seek moments of contestation and divergent analytical deductions. Advancing toward a more participatory research design, involving open-ended questioning and collaborative theorization on race and racial discrimination, is equally necessary (Salma and Temuri, 2024). When approached in this way, photo-elicitation holds significant potential to deepen our understanding of how people in refugee encampments experience the spatial dynamics of racial discrimination.

## 5 Conclusion —*locale* matters

Although the methodological application of photo-elicitation with refugees has seen a significant increase (Lenette and Boddy, 2013), scholars have not previously conducted data collection with refugees housed in tent-based refugee encampments, nor on racial discrimination. I argue that we need to pay attention to the *locale* and circumstances of the built environment of how refugees are accommodated (tents, containers, other lightly built constructions, heavier constructions, repurposed buildings, and so forth) and how these circumstances and temporalities of dwelling play into both their perceptions and practices of photographing as an embodied practice and levels of engagement.

In this article, I showed the methodological potentials and limitations of seeing spatial dimensions of racial discrimination in the refugee tent city of Bensheim through different photo-based methods. In my understanding, photographs are not merely illustrative tools depicting social realities; rather, photographs shape—and contribute to—our approaches, concepts, and research findings through which we investigate and interpret lived realities. By tracing the successive assumptions in the use of different photo-based methods, I explicitly aimed at the visualization of refugees’ everyday experiences, with particular attention to the concrete materialities of housing and their expected portrayal of spatial dimensions of racial discrimination. As shown in this case study, conceptual limitations stem from assumptions rooted in academic debates on race and ethnicity, from researchers’ preconceived sociospatial realities embedded in other studies on refugee encampments, and from the extent of empowerment grounded in “participatory research.” “Making pictures may be a valuable part of a process to improve the situation of underrepresented or marginalized people, but there is nothing intrinsically or automatically empowering in using pictures” (Pauwels, 2015, p. 108). Self-produced photographs by refugee respondents have gained popularity for their potential to center participant agency. The strength of the auto-driven photo-elicitation is demonstrated in that female refugees “unseen” from the aerial and researchers’ perspectives contributed to understanding the multi-layered spatial dimensions of racial discrimination, both attributed and self-attributed. In this study, photographic images produced by respondents surpassed verbal interviews both technically and in terms of content (Pauwels, 2015, p. 98). However, the photo-elicitation also raised critical questions regarding power dynamics, representation, and methodological and reflexive depth (Pink, 2001), which were difficult to address in a multi-ethnic refugee encampment. While photo-based approaches can provide female perspectives (Salma and Temuri, 2024), they can also reproduce dominant visual tropes, categorizations of other inhabitants, and even overlook the spatial embeddedness of experiences, especially in contexts marked by *spatially enacted classificatory systems*. Far from universally showing “their truth” (Weber, 2019), I argue that we should comprehend these photo-based methods as subjective and embodied acts of knowledge production, which have the potential to transcend researchers’ situated knowledge and assumptions grounded in both methodologies and concepts, especially when dealing with racial discrimination.

## Data Availability

The original contributions presented in the study are included in the article/supplementary material. Further inquiries can be directed to the corresponding author.

## Footnotes

1 Although visual qualitative research on refugees and minorities has increased, studies on their perceptions of spatial dimensions (Lynn and Lea, 2005; Milne and Muir, 2020), the built environment and architecture (Misselwitz et al., 2022), in interplay with their mental-health and wellbeing (Glaw et al., 2017; Mehran et al., 2021; Pain, 2012), are very disparate, without either a unifying methodology or shared conceptualizations of racism or racial discrimination.

2 *Locale* references for photographing in a specific place, where the location itself plays an integral role in the final image.

3 Spatial concepts to describe tent-based refugee camps mainly derive from empirical findings in the Global South−such as large scale “city-camps” of Dadaab crystallizing new identities and “hybrid socialization” (Agier et al., 2002, p. 336), or tent cities in Jordan as forms of urban dwelling (Dalal, 2022).

4 Across Hesse, both the initial reception center in Gießen and secondary refugee accommodation sites in Marburg-Bidenkopf, Marburg Stadtallendorf, Wetzlar, Limburg, Kassel-Calden airport, Darmstadt, and Bensheim consist partially or entirely of tent structures.

5 The language is revealing, as Kreichauf (2018) points out; unlike terms such as “camp,” “accommodation” or “center,” the labels “tent camp” or “tent city” evoke fewer associations with containment and surveillance.

6 I refer to the refugees who were involved directly in the research as “respondents” to indicate their level of engagement in this study. The term “participants” would imply a deeper involvement—such as contributing to the research design, influencing the study’s outcomes, or engaging in a potentially activist, goal-oriented process.

7 The go-along method, introduced by Kusenbach (2003), involves accompanying participants as they move through their everyday environments in order to understand how they make sense of their sociomaterial surroundings *in situ*.

8 On the difficulties and potential conflicts arising from different framings through translation, as well as the dependencies inherent in the translation process (see Baker, 2006).

9 With the respondents’ and GRC workers’ approval, we recorded the interviews and subsequently transcribed the German translations provided by the GRC workers for analysis. I rewrote the transcribed quotations from indirect speech to direct language for this text to create better readability of the statements.

10 We made a formal agreement with the respondents that we would not disclose their identities nor publish photographs that depict them, family members or other residents of the tent city. Names, ages, and details of places have therefore been made up.

11 *Minijobs* are an official German category of work, which allow people to earn up to a limited amount per month.

12 All the respondents also submitted digital images they took with their mobile phones. This did not produce a series or number of photographs comparable with the analog photographs taken with the disposable cameras, as they differ technically, contextually and in terms of intention and motivation.

13 According to the Dublin III regulation No. 604 by the European Union [2013, chapter III, art.7 (2)], refugees stay in the country where they first apply for asylum or are first registered.

14 Here and elsewhere, I use the inverted commas around “young Muslim men” to signify the artificial, ideational nature of this as a homogeneous group in the imaginaries of the authorities.

## References

[ref1] Agamben G. (1998). Homo sacer: sovereign power and bare life. Stanford: Stanford University Press.

[ref2] Agier M. Nice R. Wacquant L. (2002). Between war and city: towards an urban anthropology of refugee camps. Ethnography 3, 317–341. doi: 10.1177/146613802401092779

[ref3] Alexopoulou M. (2019). “‘Ausländer’ – a racialized concept? ‘Race’ as an analytical concept in contemporary German immigration history” in Who can speak and who is heard/hurt? Facing problems of race, racism, and ethnic diversity in the humanities in Germany. eds. Arghavan M. Hirschfelder N. Kopp L. Motyl K. (Bielefeld: transcript-Verlag), 45–68.

[ref4] Alfter S. (2023). Endstation Bergstraße: was Geflüchtete in einer Bensheimer Zeltstadt erleben. Mannheimer Morgen. Available online at: https://www.mannheimer-morgen.de/metropolregion_artikel,-metropolregion-endstation-bergstrasse-was-gefluechtete-in-einer-bensheimer-zeltstadt-erleben-_arid,2050231.html?&npg (Accessed May 17, 2023).

[ref5] Attia I. (2012). Privilegien sicher, nationale Identität revitalisieren. J. Psychol. 21, 1–31. doi: 10.23668/psycharchives.14808

[ref6] Baker M. (2006). Translation and conflict. A narrative account. London: Routledge.

[ref7] Balibar É. (2004). We, the people of Europe. Reflections on transnational citizenship. Princeton: Princeton University Press.

[ref8] Balibar É. Wallerstein I. M. (2019). Rasse, klasse, nation: ambivalente identitäten. 7th Edn. Hamburg: Argument Verlag.

[ref9] Barry C. (2025). “Conversation with the specters of the camp. Thinking refugee resistance through the Lens of the mass strike and convivial futurity” in The coloniality of human hierarchy: disrupting racial capitalism & fostering transnational solidarity. eds. Abay R. A. Garba F. M. Ihring I. (Lanham: Lexington Books).

[ref10] Barwick-Gross C. Chollet J. Kulz C. (2025). Researching urban diversity and the (re)production of whiteness: reflections on the purchase and challenges of sensory methods. Front. Sociol. 10, 1–12. doi: 10.3389/fsoc.2025.1512271 PMC12093772 40401043

[ref11] Belina B. Michel B. (2019). Raumproduktionen. Beiträge der Radical Geography: eine Zwischenbilanz. 4th Edn. Münster: Westfälisches Dampfboot.

[ref12] Bojadzijev M. (2015). “Rassismus ohne Rassen, fiktive Ethnititäten und das genealogische Schema. Überlegungen zu Étienne Balibars theoretischem Vokabular für eine kritische Migrations- und Rassismusforschung” in Schlüsselwerke der Migrationsforschung: Pionierstudien und Referenztheorien. eds. Reuter J. Mecheril P. (Wiesbaden: Springer VS).

[ref13] Bormann C. Werner F. (2024). “Urbane Räume: Ankunftsräume für Geflüchtete und ihre Funktionen” in Flucht, Raum, Forschung: Einführung in die raumsensible FluchtMigrationsforschung. eds. Werner F. Piechura P. Bormann C. Breckner I.. 5th ed (Wiesbaden: Springer VS). Frankfurt: Suhrkamp

[ref14] Bourdieu P. Adamson M. (1990). In other words. Essays towards a reflexive sociology. Stanford: Stanford University Press.

[ref15] Bourdieu P. Wacquant L. (2022). Reflexive anthropologie. 5th Edn. Frankfurt: Suhrkamp.

[ref16] Brantner C. (2018). New visualities of space and place: mapping theories, concepts and methodology of visual communication research on locative media and geomedia. Westminster Pap. Commun. Cult. 13, 14–30. doi: 10.16997/wpcc.290

[ref17] Brown T. L. (2019). Racialized architectural space: a critical understanding of its production, perception and evaluation. Architecture 15, 1–32. doi: 10.14324/111.444.amps.2019v15i3.001

[ref18] Buhr F. (2021). “Migrants' mental maps: unpacking inhabitants' practical knowledge in Lisbon” in Visual methodologies in migrant studies. New possibilities, theoretical implications, and ethnical questions. eds. Niekilska-Sekula K. Desille A. (Cham: Springer).

[ref19] Bulley D. (2014). Inside the tent: community and government in refugee camps. Secur. Dialogue 45, 63–80. doi: 10.1177/0967010613514788

[ref20] Castro Varela M. Mecheril P. (2016). “Die Dämonisierung des Anderen. Einleitende Bemerkungen” in Die Dämonisierung der Anderen. Rassismuskritik der Gegenwart. eds. Castro Varela M. Mecheril P. (Bielefeld: transcript-Verlag).

[ref21] Clair M. Denis J. S. (2015). “Racism, sociology of” in International encyclopedia of the social and behavioral sciences. eds. Smelser N. J. Baltes P. B. (Amsterdam and Heidelberg: Elsevier).

[ref22] Dalal A. (2022). From shelters to dwellings. The Zaatari Refugee Camp. Bielefeld. Available online at: https://tufind.hds.hebis.de/Record/HEB508884578

[ref23] De Certeau M. (1985). “Practices of space” in On Signs. ed. Blonsky M. (Baltimore: Johns Hopkins University Press).

[ref24] Delaney D. (2002). The space that race makes. Prof. Geogr. 54, 6–14. doi: 10.1111/0033-0124.00309

[ref25] den Van Scott L. J. (2018). Visual methods in ethnography. J. Contemp. Ethnogr. 47, 719–728. doi: 10.1177/0891241618806972

[ref26] Dizdar D. Hirschauer S. Paulmann J. Schabacher G. (2021). “Humandifferenzierung. Disziplinäre Perspektiven und transdisziplinäre Anschlüsse” in Humandifferenzierung: Disziplinäre Perspektiven und empirische Sondierungen. eds. Dizdar D. Hirschauer S. Paulmann J. Schabacher G. (Baden-Baden: Velbrück).

[ref27] El-Kayed N. Hamann U. (2018). Refugees’ access to housing and residency in German cities: internal border regimes and their local variations. SI 6, 135–146. doi: 10.17645/si.v6i1.1334

[ref28] El-Tayeb F. (2011). European others: queering ethnicity in postnational Europe. Minnesota: University of Minnesota Press.

[ref29] El-Tayeb F. (2016). Undeutsch. Die Konstruktion des Anderen in der postmigrantischen Gesellschaft. Bielefeld: transcript-Verlag.

[ref30] European Union (2013). Regulation (EU) no 604/2013 of European Parliament and of the council of 26 June 2013 establishing the criteria and mechanisms for determining the member state responsible for examining an application for international protection lodged in one of the member states by a third-country national or a stateless person (recast). Available online at: https://eur-lex.europa.eu/legal-content/EN/TXT/?uri=CELEX%3A32013R0604 (Accessed October 12, 2024).

[ref31] Farman J. (2010). Mapping the digital empire: Google earth and the process of postmodern cartography. New Media Soc. 12, 869–888. doi: 10.1177/1461444809350900

[ref32] FAZ (2015): Hessen, Land der Zelte. [Hesse, land of tents]. Available at: https://www.faz.net/aktuell/rhein-main/hessen/hessen-zeltstaedte-fuer-fluechtlinge-in-darmstadt-und-bensheim-13753509.html (Accessed October 13, 2023).

[ref33] Ganster M. Kaufhold M. (2023). Frankfurt: Wie Flüchtlinge in Bensheim in einer Zeltstadt ausharren. Available online at: https://www.faz.net/aktuell/rhein-main/region-und-hessen/frankfurt-wie-fluechtlinge-in-bensheim-in-einer-zeltstadt-ausharren-18683236.html (Accessed June 4, 2025).

[ref34] Gentzel P. Wimmer J. (2025). Restricted but satisfied: google maps and agency in the mundane life. Convergence 30, 1041–1057. doi: 10.1177/13548565231205869

[ref35] Gerritz U. (2024). Flüchtlingssituation im Kreis Bergstraße entspannt sich - vorerst. Available online at: https://www.hessenschau.de/gesellschaft/fluechtlingssituation-im-kreis-bergstrasse-entspannt-sich---vorerst-v1,fluechtlinge-bergstrasse-100.html (Accessed July 14, 2023).

[ref36] Glaw X. Inder K. Kable A. Hazelton M. (2017). Visual methodologies in qualitative research. Int J Qual Methods 16, 1–8. doi: 10.1177/1609406917748215

[ref37] Goodfriend S. (2021). A street view of occupation: getting around Hebron on Google maps. Vis. Anthropol. Rev. 37, 225–245. doi: 10.1111/var.12247

[ref38] Grbac P. (2013). Civitas, polis, and urbs: Reimagining the refugee camp as a city,” Working Paper Series No. 96. Oxford: University of Oxford: Refugee Studies Centre.

[ref39] Hall S. (2013). “The work of representation” in Representation. eds. Hall S. Evans J. Nixon S. (London: Sage).

[ref40] Haraway D. (1988). Situated knowledges: the science question in feminism and the privilege of partial perspective. Feminist Stud. 14, 575–599. doi: 10.2307/3178066

[ref41] Harper D. (2012). Visual Sociology. New York, NY: Routledge.

[ref42] Herslund L. Paulgaard G. (2021). Refugees’ encounters with Nordic rural areas-darkness, wind and “hygge”! Front. Sociol. 6, 1–11. doi: 10.3389/fsoc.2021.623686 PMC8022831 33869575

[ref43] Hirschauer S. (2021). Menschen unterscheiden. Grundlinien einer Theorie der Humandifferenzierung. Distinguishing humans. The outline of a theory of human differentiation. ZfS 50, 155–174. doi: 10.1515/zfsoz-2021-0012

[ref44] Hirschauer S. (2023). “Unterscheiden und Zuordnen. Skizze einer Theorie der Humandifferenzierung” in Mobilität und Differenzierung. Zur Konstruktion von Unterschieden und Zugehörigkeiten in der europäischen Neuzeit. eds. Panter S. Paulmann J. Weller T. (Göttingen: Vandenhoeck & Ruprecht).

[ref45] HMWEVW (2022). Hinweise zu den bauaufsichtlichen Anforderungen für die Unterbringung von Flüchtlingen und Asylbegehrenden. Stand März 2022. Available online at: https://wirtschaft.hessen.de/sites/wirtschaft.hessen.de/files/2021-07/merkblatt_erstunterkuenfte_19_10_2015-final.pdf (Accessed June 21, 2025).

[ref46] Humpage L. Fozdar F. Marlowe J. Hartley L. (2019). Photovoice and refugee research: the case for a ‘layer’ versus ‘labels’ approach to vulnerability. Res. Ethics 15, 1–16. doi: 10.1177/1747016119864353

[ref47] International Women Space (2023). Break isolation group. By refugee women, for refugee women. Available at: https://iwspace.de/break-isolation/ (Accessed July 4, 2025).

[ref48] Kappelhof J. W. S. De Leeuw E. D. (2019). Estimating the impact of measurement differences introduced by efforts to reach a balanced response among non-western minorities. Sociol. Methods Res. 48, 116–155. doi: 10.1177/0049124117701474

[ref49] Kreichauf R. (2018). From forced migration to forced arrival: the campization of refugee accommodation in European cities. CMS 6, 7–22. doi: 10.1186/s40878-017-0069-8, 29607293 PMC5874268

[ref50] Kusenbach M. (2003). Street phenomenology: the go-along as ethnographic research tool. Ethnography 4, 455–485. doi: 10.1177/146613810343007

[ref51] Lee J. Ingold T. (2006). “Fieldwork on foot: perceiving, Routining, socializing” in Locating the field. Space, place and context in anthropology. eds. Coleman S. Collins P. (Oxford: Berg).

[ref52] Lefebvre H. (1991). The production of space. Oxford: Blackwell Publishing.

[ref53] Lenette C. Boddy J. (2013). Visual ethnography and refugee women: nuanced understandings of lived experiences. Qual. Res. J. 13, 72–89. doi: 10.1108/14439881311314621

[ref54] Lipsitz G. (2007). The racialization of space and the spatialization of race. Theorizing the hidden architecture of landscape. Landsc. J. 26, 10–13. doi: 10.3368/lj.26.1.10

[ref55] Löw M. (2008). The constitution of space. The structuration of spaces through the simultaneity of effect and perception. Eur. J. Soc. Theory 11, 25–49. doi: 10.1177/1368431007085286

[ref56] Löw M. (2023). Über die Einhegung von Race in Ethnizität. Eine feministische Intervention. Berl. J. Soziol. 33, 33–42. doi: 10.1007/s11609-023-00495-z

[ref57] Lynn N. Lea S. J. (2005). Through the looking glass: considering the challenges visual methodologies raise for qualitative research. Qual. Res. Psychol. 2, 213–225. doi: 10.1191/1478088705qp039oa

[ref58] Malkki L. (2002). News from nowhere. Mass displacement and globalized ‘problems of organisation’. Ethnogr. 3, 351–360. doi: 10.1177/146613802401092797

[ref59] Marcucci O. (2024). Racial matching in qualitative interviews: integrating ontological, ethical, and methodological arguments [43 paragraphs]. Forum Qualit. Sozialfor. Forum:Qualit. Soc. Res. 25:2. doi: 10.17169/fqs-25.1.4048

[ref8004] Maria (2023). Respondent-generated Photo-documentation in the tent city ofBensheim.

[ref60] Martiniello M. (2017). Visual sociology approaches in migration, ethnic and racial studies. Ethnic Racial Stud. 40, 1184–1190. doi: 10.1080/01419870.2017.1295163

[ref61] Mehran N. Jumaa J. A. Lazaridou F. Foroutan N. Heinz A. Kluge U. (2021). Spatiality of social stress experienced by refugee women in initial reception centers. J. Int. Migr. Integr. 23, 1685–1709. doi: 10.1007/s12134-021-00890-6

[ref62] Milne E. Muir R. (2020). “Photovoice: a critical introduction” in The sage handbook of visual research methods. eds. Pauwels L. Mannay D.. 2nd ed (Los Angeles: Sage).

[ref63] Misselwitz P. Dalal A. Fraikin A. Noll A. Zaripova V. (2022). Tempohomes – Untersuchung sozial-räumlicher Aneignungspraktiken von Geflüchteten in ausgewählten Berliner Gemeinschaftsunterkünften. Berlin: Universitätsverlag der TU Berlin.

[ref64] Moskal M. (2019). “Visual methods in research with migrant and refugee children and young people” in Handbook of research methods in health social sciences. ed. Liamputtong P. (Singapore: Springer).

[ref65] Olmos-Vega F. M. Stalmeijer R. E. Varpio L. Kahlke R. (2023). A practical guide to reflexivity in qualitative research: AMEE guide no. 149. Med. Teach. 45, 241–251. doi: 10.1080/0142159X.2022.2057287, 35389310

[ref66] Pain H. (2012). A literature review to evaluate the choice and use of visual methods. Int J Qual Methods 11, 303–319. doi: 10.1177/160940691201100401

[ref67] Pauwels L. (2015). ‘Participatory’ visual research revisited: a critical-constructive assessment of epistemological, methodological and social activist tenets. Ethnography 16, 95–117. doi: 10.1177/1466138113505023

[ref68] Pink S. (2001). More visualising, more methodologies: on video, reflexivity and qualitative research. Sociol. Rev. 49, 586–599. doi: 10.1111/1467-954X.00349

[ref69] Pink S. (2008). Mobilising visual ethnography: making routes, making place and making images. FQS 9:36. doi: 10.17169/fqs-9.3.1166

[ref70] Poole D. (2005). An excess of description: ethnography, race and visual technologies. Annu. Rev. Anthropol. 34, 159–179. doi: 10.1146/annurev.anthro.33.070203.144034

[ref71] Prieto-Blanco P. (2021). “Afterword: visual research in migration. (in)visibilities, participation, discourses” in Visual methodology in migrant studies: new possibilities, theoretical implications, and ethical questions. eds. Nikielska-Sekula K. Desille A. (Cham: Springer).

[ref72] Richard V. M. Lahman M. K. (2015). Photo-elicitation: reflexivity on method, analysis, and graphic portraits. Int. J. Res. Method Educ. 38, 3–22. doi: 10.1080/1743727X.2013.843073

[ref73] Sadeghi S. (2019). Racial boundaries, stigma, and the re-emergence of “always being foreigners”: Iranians and the refugee crisis in Germany. Ethnic Racial Stud. 42, 1613–1631. doi: 10.1080/01419870.2018.1506145

[ref74] Salma J. Temuri H. (2024). The art and politics of participant-driven photo-elicitation with Muslim immigrant older women. Int J Qual Methods 23, 1–15. doi: 10.1177/16094069241241997

[ref75] Santos B. (2018). “The end of the cognitive empire” in The coming of age of epistemologies of the south (Durham & London: Duke University Press).

[ref76] Sanyal R. (2012). Refugees and the city: an urban discussion. Geogr. Compass 6, 633–644. doi: 10.1111/gec3.12010

[ref77] Schäfer P. (2015). Das Flüchtlingswohnheim. Raumcharakter und Raumpraxis in der Gemeinschaftsunterkunft. sinnprovinz. Available online at: http://nbn-resolving.de/urn:nbn:de:bsz:352-0-292842 (Accessed August 18, 2025).

[ref78] Schwartz D. (1989). Visual ethnography: using photography in qualitative research. Qual. Sociol. 12, 119–154. doi: 10.1007/BF00988995

[ref79] Sommer V. Töppel M. (2021). “Go-Alongs in einem multimethodischen Forschungsprogramm” in Handbuch qualitative und visuelle Methoden der Raumforschung. eds. Heinrich A. J. Marguin S. Million A. Stollmann J. (Bielefeld: transcript-Verlag).

[ref80] Spiegel (2015). Platznot: Niedersachsen fordert Flüchtlingsheime in Aldi-Bauweise. DER SPIEGEL. Available online at: https://www.spiegel.de/politik/deutschland/niedersachsen-will-fluechtlingsheime-in-aldi-bauweise-a-1045104.html (Accessed January 2, 2024).

[ref81] Steets S. (2015). Der sinnhafte Aufbau der gebauten Welt. Eine Architektursoziologie. Berlin: Suhrkamp.

[ref82] Stehle M. (2006). Narrating the ghetto, narrating Europe: from Berlin, Kreuzberg to the banlieues of Paris. WPCC 3, 49–70. doi: 10.16997/wpcc.59

[ref83] Steigemann A. Misselwitz P. (2020). Architectures of asylum. Making home in a state of permanent temporariness. Curr. Sociol. 68, 628–650. doi: 10.1177/0011392120927755

[ref84] Tessitore F. (2022). The asylum seekers photographic interview (ASPI): evaluation of a new method to increase Nigerian asylum seekers’ narrative meaning-making after trauma. Psychol. Trauma 14, 66–79. doi: 10.1037/tra0000913, 34435812

[ref85] Valiquette T. Su Y. (2024). Combining photovoice and videovoice for participatory research: visual storytelling with LGBTQ+ refugees and migrants. J. Particip. Res. Methods. 5, 1–22. doi: 10.35844/001c.123790

[ref86] Wacquant L. (1989). Towards a reflexive sociology: a workshop with Pierre Bourdieu. Sociol Theory 7, 26–63. doi: 10.2307/202061

[ref87] Wacquant L. (2007). Territorial stigmatization in the age of advanced marginality. Thesis Elev. 91, 66–77. doi: 10.1177/0725513607082003

[ref88] Wacquant L. (2023). Immer Ärger mit Race. Eine Agenda für den Umgang mit einer heiklen Kategorie. Berl. J. Soziol. 33, 9–32. doi: 10.1007/s11609-023-00494-0

[ref89] Weber S. (2019). Participatory visual research with displaced persons: ‘listening’ to post-conflict experiences through the visual. J. Refug. Stud. 32, 417–435. doi: 10.1093/jrs/fey038

[ref90] Wojnicka K. Nowicka M. (2024). Unveiling racism through qualitative research: the politics of interpretation. Qual. Res. 24, 1142–1161. doi: 10.1177/14687941231216640

